# A Reevaluation of the Morphology, Paleoecology, and Phylogenetic Relationships of the Enigmatic Walrus *Pelagiarctos*

**DOI:** 10.1371/journal.pone.0054311

**Published:** 2013-01-16

**Authors:** Robert W. Boessenecker, Morgan Churchill

**Affiliations:** 1 Department of Geology, University of Otago, Dunedin, Otago, New Zealand; 2 University of California Museum of Paleontology, Berkeley, California, United States of America; 3 Department of Geology and Geophysics, University of Wyoming, Laramie, Wyoming, United States of America; 4 Program in Ecology, University of Wyoming, Laramie, Wyoming, United States of America; Team ‘Evo-Devo of Vertebrate Dentition’, France

## Abstract

**Background:**

A number of aberrant walruses (Odobenidae) have been described from the Neogene of the North Pacific, including specialized suction-feeding and generalist fish-eating taxa. At least one of these fossil walruses has been hypothesized to have been a specialized predator of other marine mammals, the middle Miocene walrus *Pelagiarctos thomasi* from the Sharktooth Hill Bonebed of California (16.1–14.5 Ma).

**Methodology/Principal Findings:**

A new specimen of *Pelagiarctos* from the middle Miocene “Topanga” Formation of southern California (17.5–15 Ma) allows a reassessment of the morphology and feeding ecology of this extinct walrus. The mandibles of this new specimen are robust with large canines, bulbous premolars with prominent paraconid, metaconid, hypoconid cusps, crenulated lingual cingula with small talonid basins, M_2_ present, double-rooted P_3_–M_1_, single-rooted P_1_ and M_2_, and a P_2_ with a bilobate root. Because this specimen lacks a fused mandibular symphysis like *Pelagiarctos thomasi*, it is instead referred to *Pelagiarctos* sp. This specimen is more informative than the fragmentary holotype of *Pelagiarctos thomasi*, permitting *Pelagiarctos* to be included within a phylogenetic analysis for the first time. Analysis of a matrix composed of 90 cranial, dental, mandibular and postcranial characters indicates that *Pelagiarctos* is an early diverging walrus and sister to the late Miocene walrus *Imagotaria downsi*. We reevaluate the evidence for a macropredatory lifestyle for *Pelagiarctos*, and we find no evidence of specialization towards a macrophagous diet, suggesting that *Pelagiarctos* was a generalist feeder with the ability to feed on large prey.

**Conclusions/Significance:**

This new specimen of *Pelagiarctos* adds to the knowledge of this problematic taxon. The phylogenetic analysis conclusively demonstrates that *Pelagiarctos* is an early diverging walrus. *Pelagiarctos* does not show morphological specializations associated with macrophagy, and was likely a generalist predator, feeding on fish, invertebrates, and the occasional warm-blooded prey item.

## Introduction

The extant walrus *Odobenus rosmarus* represents one of the most morphologically aberrant members of the Pinnipedia, and is the sole extant member of the family Odobenidae. Despite the low taxonomic diversity of walruses today, the fossil record indicates that odobenids over the past 16 Ma showed a wider variety of morphological adaptations and body sizes than at present, exploited a greater number of resources based on inferred feeding ecology, and inhabited a wider variety of marine environments including subtropical shallow marine settings [1]–[7].

Despite the extensive fossil record of walruses during the Neogene (see table 3.2 of Deméré et al., [8]), the phylogenetic relationships of walruses remain poorly understood. Non-cladistic studies have placed walruses as the sister taxon of the Otariidae (fur seals and sea lions) within a monophyletic Otarioidea ( =  Otariidae of Barnes [9]. In contrast, phylogenetic analyses of morphological data support a sister taxon relationship between walruses and phocoid pinnipeds (Desmatophocidae+Phocidae), forming the clade Phocomorpha (i.e. Odobenidae+Phocoidea; Berta and Wyss [10]). However, virtually all molecular analyses of extant pinnipeds have supported a monophyletic Otarioidea [11]–[16].

The “Imagotariinae” are a group of stem walruses considered by Barnes [9], Barnes and Raschke [1] and Kohno et al. [6] to be a subfamily of “walrus-like” pinnipeds; Deméré [2], however, found the group to be paraphyletic, a conclusion supported by additional phylogenetic analyses [2], [4], [5], [17], and to represent a stem grade of walruses ancestral to Dusignathinae and Odobeninae. “Imagotariinae” includes *Proneotherium*, *Prototaria*, *Neotherium*, *Kamtschatarctos*, *Imagotaria*, *Pelagiarctos*, *Pseudotaria*, and possibly *Pontolis* ([5]; other analyses include *Pontolis* as a dusignathine walrus; [2]). The earliest diverging walruses – *Prototaria*, *Proneotherium*, and *Neotherium* – are generally similar in size and cranial, mandibular, and dental morphology to early diverging pinnipedimorphs ( = “Enaliarctinae” of Mitchell and Tedford [18]), while geologically younger and later diverging taxa such as *Pelagiarctos* and *Imagotaria* are much larger; *Imagotaria, Pseudotaria,* and all later diverging odobenids (Dusignathinae and Odobeninae) share numerous derived cranial features, such as a transversely arched palate, a reduced pseudosylvian sulcus, and less than three roots on the M^1^ [2], [5].

**Figure 1:**
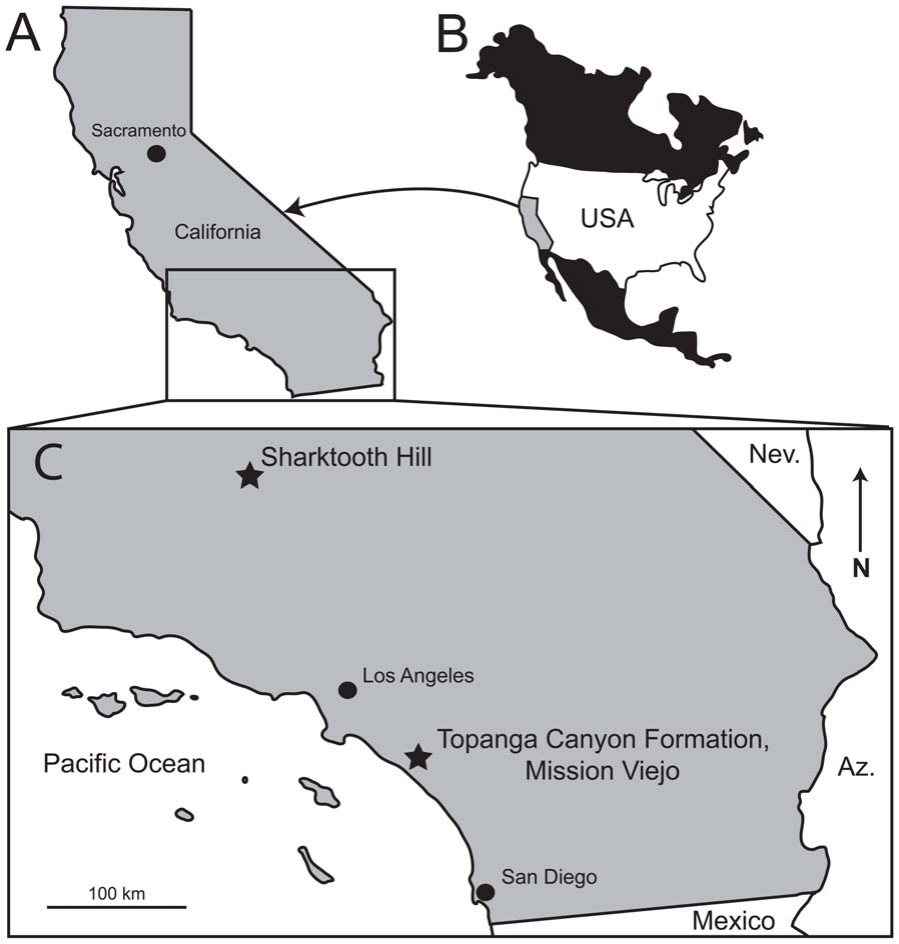
Location of fossil occurrences of *Pelagiarctos*. Map of A) California and B) North America, and C) Map of southern California showing the location of Sharktooth Hill (Round Mountain Silt) and the “Topanga” Formation. Abbreviations: Az., Arizona; Nev., Nevada.

**Figure 2:**
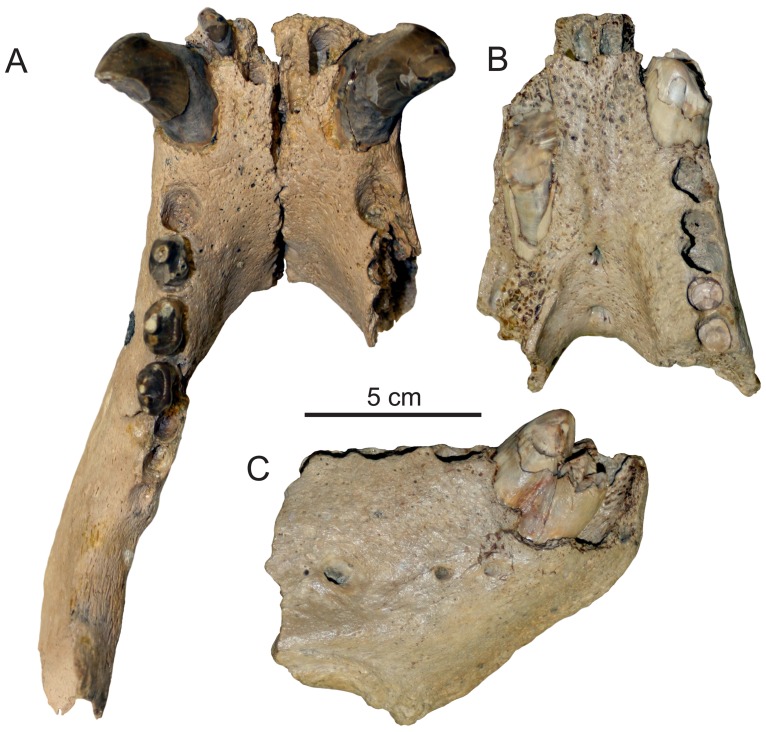
Mandibles of *Pelagiarctos*. Comparison of A) *Pelagiarctos* sp. (SDNHM 131041) in dorsal aspect, and the holotype specimen of *Pelagiarctos thomasi* (LACM 121501) in B) dorsal and C) lateral aspect.

*Pelagiarctos thomasi* was described on the basis of an isolated and fragmentary pair of fused mandibles and several isolated teeth from the middle Miocene Sharktooth Hill Bonebed in Kern County, California [19]. Due to the apparently large body size of *Pelagiarctos*, the morphology of its postcanine teeth, robust mandible, and rarity in the Sharktooth Hill Bonebed, Barnes [19] concluded that *Pelagiarctos* was a marine apex predator. *Pelagiarctos* was hypothesized to be adapted toward macrophagy and fed upon warm blooded prey such as marine birds and small marine mammals, “in addition to (or instead of) the expected diet of fishes” ([19]:9). Since the discovery of the *Pelagiarctos thomasi* holotype, only a few isolated teeth referable to the same taxon have been discovered at Sharktooth Hill ([19]; see Table 1) and we only know of a few isolated teeth that have been collected since the taxon was named by Barnes [19]; these newly collected specimens remain uncurated. Description of a new fossil (SDNHM 131041) from the “Topanga” Formation (Fig. 1) referable to *Pelagiarctos* serves to expand our knowledge of the anatomy of this poorly known walrus (Fig. 2). Although fragmentary, this new specimen offers the opportunity to include *Pelagiarctos* within a phylogenetic analysis for the first time, and to reevaluate its hypothesized feeding ecology.

**Table 1:** Table of known fossils of *Pelagiarctos*. *Pelagiarctos* sp. is from the “Topanga” Formation (This study), while all other material listed is from the Sharktooth Hill Bonebed, Round Mountain Silt (Barnes, 1988).

Specimen	Taxonomic Referral	Element
**LACM 121501**	*Pelagiarctos thomasi* holotype	symphyseal portion of mandibles, damaged I_2_, C_1_, P_3_
**LACM 123415**	*Pelagiarctos thomasi*	right P^3^ or P^4^
**LACM 38812**	*Pelagiarctos thomasi*	right P_1_
**LACM 72856**	*Pelagiarctos thomasi*	right P_3_ or P_4_
**LACM 122310**	*Pelagiarctos thomasi*	left P_3_ or P_4_
**TATE 2694**	*Pelagiarctos thomasi*	left P_1_ or right P^1^
**UCMP 93058**	*Pelagiarctos thomasi*	left P_2_
**SDNHM 131041**	*Pelagiarctos* sp.	Partial right and left dentary with left I_3_, right and left C_1_, and left P_2_–P_4_

## Materials and Methods

The new specimen of *Pelagiarctos* (SDNHM 131041) was compared with the *Pelagiarctos thomasi* holotype and referred specimens described by Barnes (Table 1; [19]), in addition to all odobenids (fossil and modern) for which mandibles are known. With the exception of *Ontocetus emmonsi* and *Kamtschatarctos sinelnikovae* (represented by casts), we examined the original fossil and modern specimens of these taxa (published and unpublished). Anatomical terminology used in this study follows Mead and Fordyce [20] for mammals.

### Phylogenetic Methods

To understand the evolutionary relationships of *Pelagiarctos* with other walruses, a phylogenetic analysis was performed using maximum parsimony in TNT [21] and Bayesian inference in MrBayes 3.1.2 [22]. Sixteen walrus taxa were included within our analysis, including representatives of the later diverging Odobeninae and Dusignathinae. Seven outgroup taxa were included for comparison: the “enaliarctine” taxa *Enaliarctos emlongi* [23] and *Pteronarctos goedertae* [9]; two members of the extinct Desmatophocidae (*Desmatophoca oregonensis* [24] and *Allodesmus gracilis* [25]); one otariid taxon (*Callorhinus ursinus*), and two phocid taxa, representing Monachinae (*Monachus tropicalis*) and Phocinae (*Erignathus barbatus*). The otariid and phocid taxa selected were chosen as they represent the earliest diverging extant lineages within their respective families and subfamilies. A complete list of specimens examined is presented within Text S1. As part of this study, we produced a matrix of 90 cranial, dental, mandibular, and postcranial characters to examine the phylogenetic position of *Pelagiarctos*. These characters are fully illustrated on Morphobank. These characters were then included within a larger dataset employing the cranial and postcranial characters of Kohno [5], resulting in a matrix of 90 morphological characters. Only minor changes were made to the original matrix, these mostly pertain to using narrower taxonomic units for coding, whenever possible using species as our terminal units, not genera, subfamilies, or families. The only exception for this rule was *Ontocetus*, in which neither author has been able to view mandibular and cranial material referable to the same species. In this case, *O. emmonsi* forms the basis of our coding for mandibular and lower dental characters, while coding of other features is largely based on that of Kohno [5]. The phylogenetic matrices for the mandible and dental characters with associated media is archived at http://morphobank.org. Characters used in this analysis were based on personal observation as well as prior phylogenetic studies [2], [9], [10], [26], and are included within Text S2. We include and code for the first time a referred mandible of *Proneotherium repenningi* (USNM 314628), which is referable based on size, similarity with *Neotherium mirum* (e.g. LACM 123000, UCMP 81665, 116018, 191874), and lack of a carnassial-like lower molar that characterizes Astoria Formation enaliarctine pinnipeds.

All parsimony analyses were run using the new technology options with 10,000 random addition replicates and an implied weighting scheme of K = 2–5. Using implied weighting allowed us to downweight characters prone to homoplasy, reducing their impact on the phylogeny [27]. Bootstrap values were calculated using symmetric resampling with 1000 replicates. Bayesian Inference analyses were run using 10 million generations, 4 chains, and a sample frequency of 1000. A conservative burn-in of 5000 was discarded. Two separate runs were created with each analysis, and a consensus of both runs was used to produce the most probable tree. Bayes factors were used to determine the morphological model of evolution employed in the analysis, with the GTR model [28] selected. Bootstrap support (BS) and Bayesian posterior probabilities (PP) are reported for all nodes.

## Results

### Systematic Paleontology

Mammalia Linnaeus, 1758.

Carnivora Bowdich, 1821.

Odobenidae Allen, 1880.

Pelagiarctos Barnes, 1988.

#### Emended diagnosis

*Pelagiarctos* is distinguished from the early diverging odobenids *Proneotherium, Neotherium*, and *Kamtschatarctos* by the following derived characters: rugose, vascularized bone at mandibular terminus; mandible that is dorsoventrally much deeper anteriorly, C_1_ with lateral sulci, inflated postcanine crowns, less prominent metaconid cusps, and medially swollen and crenulated lingual cingulum. *Pelagiarctos* differs from the later diverging odobenids *Imagotaria* and *Pontolis* in retaining the following primitive characters: double-rooted P_2–4_; metaconid cusps; sinuous ventral margin of mandible. *Pelagiarctos* is distinguished from all dusignathine and odobenine walruses in the retention of the following primitive characters: well developed paraconid, metaconid, and hypoconid cusps on postcanine crowns; double-rooted lower postcanines; lower canine with posterior crista; retention of M_2_.

### *Pelagiarctos* sp

#### Horizon and age

SDNHM 131041 was collected in 1997 by H. Lozana from SDNHM locality 4984 in Orange County, California (Fig. 1). SDNHM locality 4984 was a temporary exposure of a rock unit currently mapped as the “Topanga” Formation, exposed during construction within the city of Mission Viejo (Fig. 1). Locally, the “Topanga” Formation overlies the lower Miocene Vaqueros Formation, and is overlain by the middle-upper Miocene Monterey Formation. Detailed locality information is available on request. Howard and Barnes [29] noted that the Topanga Canyon Group is probably not an appropriate stratigraphic name to be applied to this unit in Orange County; the type section in Los Angeles County has been renamed and elevated to group status [30]. Because no stratigraphic revision of this unit has been published in the time being, we retain the current applicable name, although it will likely change in the future due to concerns addressed by Howard and Barnes [29], and we follow Whistler and Lander [31] in referring to this unit in Orange County as the “Topanga” Formation to reflect its difference from the type section of the Topanga Canyon Group in Los Angeles County. Fossil vertebrates from SDNHM locality 4984 occur in a marine bonebed within a gray to yellow, friable, medium to coarse-grained sandstone, exposed about 2 meters above a prominent oyster-rich shell bed. SDNHM locality 4984 constitutes the uppermost 3 meters of the “Topanga” Formation, below its contact with the overlying Monterey Formation. A nearby locality in the “Topanga” Formation has yielded a diverse marine vertebrate assemblage, including sharks, marine birds, pinnipeds, and odontocetes [29], [32]. Howard and Barnes [29] reported a meager marine mammal assemblage, including an archaic walrus (*Neotherium* sp.), a desmatophocid seal (*Allodesmus* sp.), an allodelphinid odontocete (cf. *Zarhinocetus errabundus*), and a kentriodontid dolphin (*Kentriodon* sp.). Although this assemblage still awaits description, all of these taxa occur within the middle Miocene (16.1–14.5 Ma) Sharktooth Hill Bonebed, suggesting a similar age of the two units [29]. At a nearby locality in the “Topanga” Formation, Aranda-Manteca et al. [33] reported a partial skull of the dugongid sirenian *Metaxytherium arctodites*; more complete material of this taxon was described from the Los Indios Member of the Rosarito Beach Formation at La Mision in Baja California. The Rosarito Beach Formation also shares a number of marine mammal taxa (aff. *Neotherium*, *Allodesmus* sp., aff. *Tiphyocetus temblorensis*, aff. *Parietobalaena* sp., aff. *Kentriodon* sp., *Liolithax* aff. *L. kernensis*, and aff. *Desmostylus* sp.) with the Sharktooth Hill assemblage [34], [35]. Several terrestrial mammals were reported from the “Topanga” Formation by Raschke [32] and Howard and Barnes [29], including an indeterminate paleomerycid, a camel (*Aepycamelus* sp.), and a horse (*Merychippus* sp.), suggesting correlation with the Barstovian North American Land Mammal Age (NALMA; [29], [32]). Further work on the terrestrial mammals of the “Topanga” Formation in this region was published by Whistler and Lander [31], who reported a much more diverse assemblage; they noted that the presence of *Copemys* and *Merychippus* indicated a slightly older age, being correlative with the early late Hemingfordian NALMA. The late Hemingfordian is approximately 17.5–15.9 Ma in age [36]. Relizian and/or Luisian benthic foraminifera and mollusks assignable to the Temblor Provincial Marine Molluscan Stage have also been reported from these localities in the “Topanga” Formation [31]. Further to the west, in the San Joaquin Hills, an andesitic lava flow at the base of the Paulerino Member of the “Topanga” Formation has been K/Ar dated at 15.8+− 1.3 Ma [37]; recalculated with revised decay constants by Whistler and Lander [31]. Taken in full, “Topanga” Formation in Orange County is probably late early to early middle Miocene in age (∼17.5–15 Ma), and is broadly correlative with the Round Mountain Silt Member of the Temblor Formation and the Los Indios Member of the Rosarito Beach Formation. Detailed locality information is available on request.

#### Referred material

SDNHM 131041, a pair of partial associated left and right mandibles, preserving left I_3_, left and right C_1_, left P_2–4_ and partial root of right P_2_. SDNHM 131041 was collected from SDNHM locality 4984 in the middle Miocene “Topanga” Formation. Because the left and right mandibles were found in close association, and tightly articulate at the symphysis when placed together (Fig. 2a), they represent one individual.

### Description of SDNHM 131041

#### Mandible

**Figure 3:**
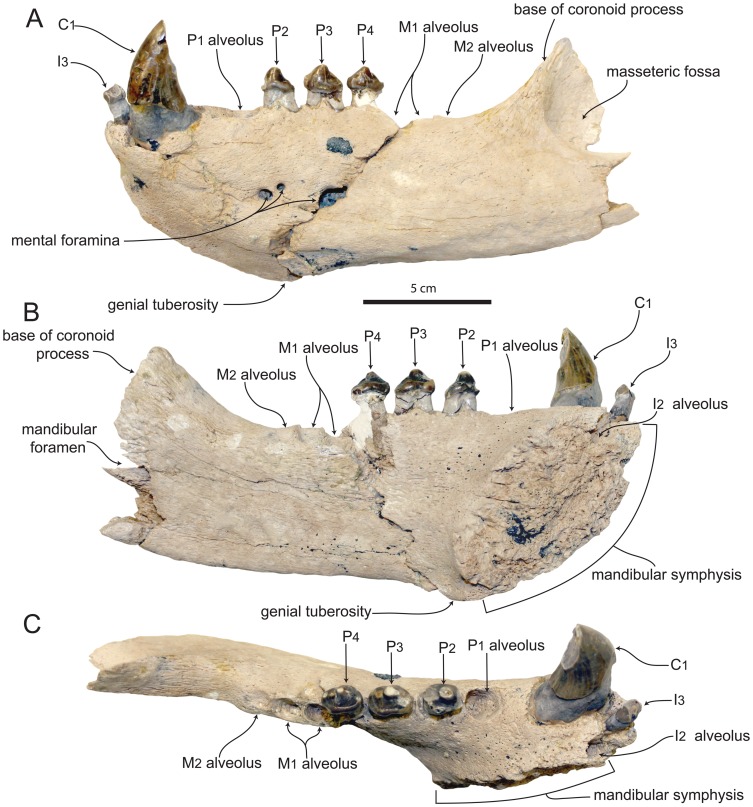
Left mandible of SDNHM 131041, *Pelagiarctos* sp. SDNHM in A) lateral, B) medial, and C) dorsal aspect.

**Figure 4:**
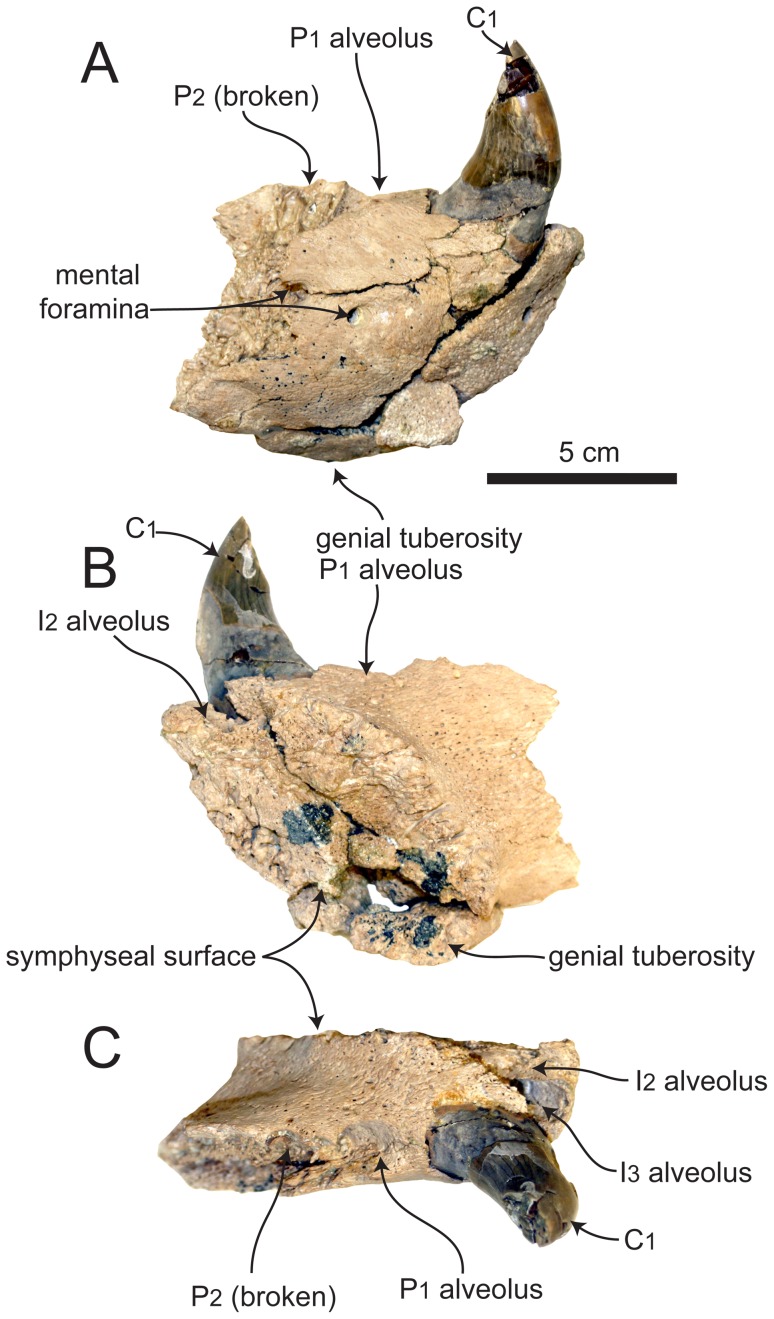
Right mandible of SDNHM 131041, *Pelagiarctos* sp. Mandible of SDNHM 131041 in A) lateral, B) medial, and C) dorsal aspect.

The referred specimen includes portions of the left and right mandibles (Fig. 2, 3, 4). The more incomplete right mandible is only preserved anterior to the P_3_, and there is some slight breakage of the dorsal portion medial to the right canine. The less damaged left mandible is missing most of the ascending ramus, and just the anteriormost portion of the coronoid process is preserved (Fig. 4). Unless stated otherwise, the description is based on the left mandible. Measurements of SDNHM 131041 are presented in Table 2.

**Table 2:** Measurements of *Pelagiarctos* sp., SDNHM 131041.

Variable	Measurement in cm
Total length (as preserved)	20.57
Anterior tip to base of ascending ramus	15.33
Greatest length of mandibular symphysis	8.78
Anteroposterior length of symphysis	7.38
Depth of mandibular symphysis	4.83
Depth of ramus at C1	5.41
Depth of ramus at P1	6.46
Depth of ramus at P2	6.95
Depth of ramus at P3	6.56
Depth of ramus at P4	6.45
Depth of ramus at M1	5.48
Depth of ramus at M2	5.46
Length of postcanine tooth row	8.98
Length of C1–P1 diastema	0.86
Anterior tip to M1	10.86
M1 to base of ascending ramus	1.76
M2 to base of ascending ramus	1.18
Width across canines (apical)	8.83
Width across canines (basal)	6.43
Transverse width of ramus at P1	3.65
Transverse width of ramus at P2	4.40
Transverse width of ramus at P3	2.71
Transverse width of ramus at P4	2.21
Transverse width of ramus at M1	2.26
Transverse width of ramus at M2	2.24
I3 height/width	0.64/0.84
Canine height (right/left)	4.15/419
Canine anteroposterior length (right/left)	2.6/2.96
P2 crown height/width/length	0.85/1.06/1/31
P3 crown height/width/length	0.97/1.03/1.52
P4 crown height/width/length	0.86/1.02/1/44

The mandibles are robust, dorsoventrally deep, and transversely wide. When in articulation, the anterior portions of the mandibles are transversely widest at the position of the canines (Fig. 2A). In lateral aspect, the dorsal and ventral margins of the horizontal ramus are nearly parallel. The dorsal margin of the coronoid process meets the horizontal ramus at an angle of 130°. Although most of the posterior portion of the mandible is missing, what is preserved of the anteriormost portion of the masseteric fossa indicates it was deep and sharply defined anteriorly. The medial surface of the coronoid process and horizontal ramus are generally flat. Along the long axis of the horizontal ramus, the vertical orientation changes so that near the position of the M_1_, the medial surface of the ramus faces slightly ventromedially, while further anterior at the position of the P_2_, the medial surface faces slightly dorsomedially. On the medial surface of the horizontal ramus there is a broad shelf immediately adjacent to the mandibular symphysis.

The symphyseal surface is rugose and pitted, but unlike the holotype specimen, the mandibles are not firmly ankylosed at the mandibular symphysis (Fig. 2A, 3B). In medial aspect, the symphyseal surface is oval-shaped, with convex anterior and posterior margins. The bone surface at the anterior end of the mandibles is rough and vascularized, with many small foramina, and most evident near the mandibular symphysis. Around the base of the canines, the lateral surface of the mandible bulges out around the canine root. Anteromedial to this, a slight medial keel exists adjacent to the mandibular symphysis. Adjacent to the symphysis and below the I_3_, a large foramen occurs on each mandible, as in the holotype specimen.

The lateral surface of the horizontal ramus is slightly convex. Two large mental foramina occur on the lateral surface of the left mandible below the P_2_, and a third large mental foramen occurs below the P_3_. On the right mandible, a small single mental foramen occurs below the P_1_. A well-developed genial tuberosity is situated at the posteroventral termination of the mandibular symphysis, although it does not sit on a ventral ridge as in the holotype specimen. Along the postcanine tooth row, adjacent alveoli and tooth roots are separated by small triangular extensions of bone.

#### Lower dentition

**Figure 5:**
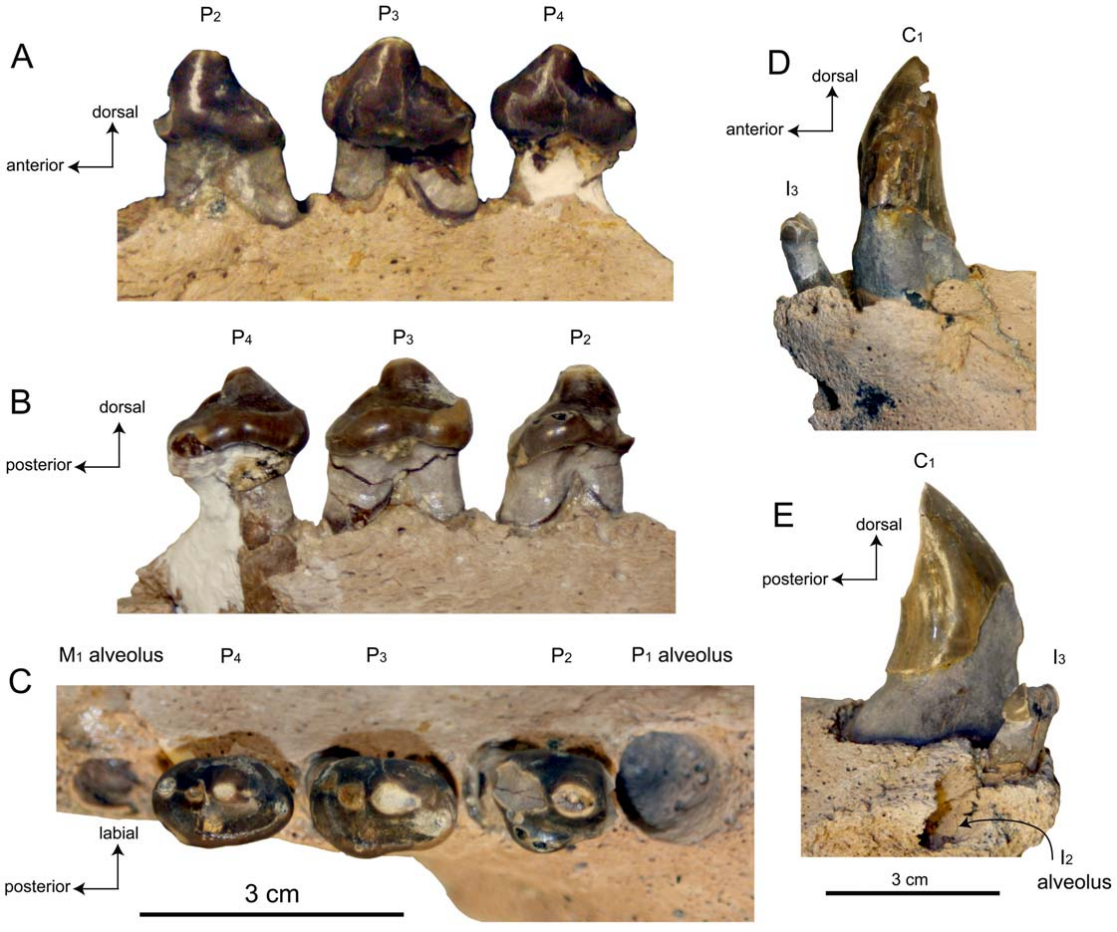
Lower dentition of SDNHM 131041, *Pelagiarctos* sp. Lower premolars in A) labial, B) lingual, and C) occlusal aspect. The M_1_ and P_1_ alveoli, and C_1_ and I_3_ are depicted in D) labial and E) mesial aspect.

On all teeth preserved, the basal margin of the crown is elevated nearly 10 mm above the alveolar margin, ranging from 8–10 mm on the P_2–3_ and nearly 20 mm on the anterior portion of the canine (Fig. 5). The I_2_ is missing, but its alveolar morphology indicates it was small with a cylindrical, anterodorsally oriented root. The I_2_ alveolus is medially confluent with the symphyseal surface, and there is no medial wall of bone preserved between the alveolus and the symphysis, indicating the lower second incisors were closely appressed; there are no I_1_ alveoli. The I_2_ alveolus is positioned posteromedially to the I_3_, resulting in a posteriorly V-shaped incisor alveolar row as in many early diverging odobenids and otariids. The left I_3_ is preserved, and is a peg-like tooth with a small, bulbous crown and smooth enamel. The crown is roughly circular in occlusal aspect, and set on a column-like root. The crown is obliterated by two anteromedial and posterolateral wear facets, formed by occlusion with the I^2^ and I^3^, respectively.

Both left and right C_1_ are present and well preserved. The canines are robust, vertically oriented, and slightly posteriorly curved (Fig. 5D, E). The crowns are conical and laterally compressed with an oval-shaped cross section, and thick, smooth enamel. There are two large wear facets on the crowns: a small anteromedial wear facet on the base of the crown from occlusion with the I^3^, and a large, posterior facing, oval-shaped apical wear facet from occlusion with the C_1_. The canines have shallow, longitudinal sulci on the lateral surface of the root, like the holotype of *Pelagiarctos thomasi* (Fig. 2, 5D, E). The sulci terminate below the crown, and there are no sulci on the medial surfaces of the canine roots.

**Figure 6:**
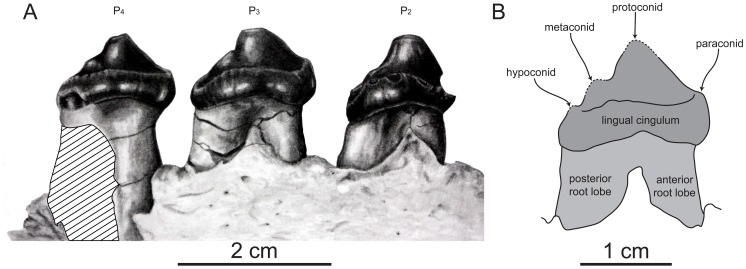
Dentition of SDNHM 131041, *Pelagiarctos* sp. A) Illustration of lower premolars in lingual aspect. B) line drawing of P_2_ in lingual aspect, showing cusp homologies.

Although both first lower premolars are missing, their alveoli are well preserved and indicate that the P_1_ had a single, cylindrical root with a circular cross-section (Fig. 5C). The P_1_ alveolus is notably wider than any other postcanine alveolus. The left P_2_ (Fig. 5A–C, 6) appears to be either double-rooted or have a bilobate root, with a longitudinal sulcus on the lateral side, and a stronger sulcus on the medial surface. The broken right P_2_ preserves root fragments in their alveoli, and no bony septum separates the roots, indicating the right P_2_ was bilobate. In occlusal aspect, the P_2_ crown is oval shaped. The P_2_ has a large principal cusp (protoconid), while the anterior cusp (paraconid) and posterior cusp (hypoconid) morphology is unknown because of two large wear facets on the anterior and posterior edges of the crown (Fig. 5A, B). A faint labial cingulum is developed along the posterolabial margin of the crown. The posterior wear facet is so extensive that it is not clear if a metaconid was present. A third apical wear facet occurs at the tip of the protoconid. A strong lingual cingulum is present, with at least three cuspules ( = crenations of Deméré and Berta [38]), although these are slightly worn. The cingulum is thickest posteromedially, where it forms a slight shelf and concavity, probably homologous to the talonid basin. The base of the crown is dorsally arched lingually and labially.

The P_3_ (Fig. 5A–C, 6) root is double-rooted above the alveolar margin, and the root lobes appear to be widely divergent. The base of the P_3_ crown is also dorsally arched lingually and labially, and the crown itself is bulbous. The crown of the P_3_ is slightly anteroposteriorly longer than the P_2_, and oval shaped in occlusal aspect. The protoconid cusp is very prominent, and there is a minute and worn paraconid cusp is present near the base of the crown and anterior to the protoconid. The tip of the protoconid has an apical wear facet; a blunt longitudinal crista connects the protoconid and paraconid. A small and worn metaconid cusp is located on the blunt posterior crista of the protoconid; the hypoconid cusp is positioned posterior and slightly labial to the metaconid, and is also worn. The labial surface is convex, with smooth enamel. A faintly developed labial cingulum is present posterolabially, which merges posteriorly with the hypoconid; anteriorly, the labial cingulum merges into a smooth surface. On the lingual surface there is a well developed lingual cingulum with fine cuspules along the crest. Between the protoconid and the cingulum is a small posterolingual shelf-like talonid basin. Due in part to the well-developed cingulum, the crown bears a posteromedial extension. A poorly developed labial cingulum occurs on the posterolabial portion of the crown, and terminates posterior to the midpoint of the crown.

Although the P_4_ (Fig. 5A–C, 6) root is damaged, the anterior root lobe is present and cylindrical, and a fragment of the posterior root lobe is preserved, clearly indicating a double-rooted condition for this tooth. The basal margin of the crown is dorsally arched lingually and labially, as in the P_2_ and P_3_ crowns. The labial surface of the crown is convex with smooth enamel, and there is a poorly defined and discontinuous labial cingulum that is positioned posterolabially. The labial cingulum extends anteriorly from the hypoconid, and extends roughly 3/4 of the way to the paraconid cusp. The bulbous crown is oval-shaped in occlusal view, and the medial surface is strongly convex. The P_4_ crown is transversely narrower than the P_3_ crown. The crown has a strong protoconid, and a small paraconid is developed on the anterior portion of the crown; a blunt longitudinal crista connects the paraconid and protoconid. A small accessory cusp occurs on the crest just anterior to the protoconid wear facet. A small metaconid cusp occurs on the posterior crista, and the hypoconid cusp occurs slightly labially to this crista. The tips of the paraconid, protoconid, metaconid, and hypoconid cusps have small circular apical wear facets (Fig. 5C). The lingual cingulum of the P_4_ is rough and bears many small cuspules (Fig. 5B, C, 6A); the cuspules are more strongly developed posterolingually on the cingulum. The cingulum defines a slight talonid and trigonid basin, and is transversely wider anterolingually.

Both M_1_ and M_2_ are missing (Fig. 2, 3). Three alveoli occur posterior to the P_4_; because most other pinnipedimorphs bearing a second lower molar show a tendency for the M_2_ (rather than the M_1_) to possess a single-rooted morphology (i.e. *Allodesmus*, *Enaliarctos*, *Imagotaria*, *Pontolis* and *Pteronarctos*) the first two molar alveoli are identified as the M_1_ alveoli, and the third alveolus is identified as a single alveolus for the M_2_. The M_1_ alveoli are deep and separated by a well-developed bony septum, indicating widely divergent root lobes. The M_2_ alveolus is shallow, and along with the relatively long root exposure above the alveolar margin in SDNHM 131041, suggests that the root of this tooth may have been primarily embedded in the gums.

### Comparisons

**Figure 7:**
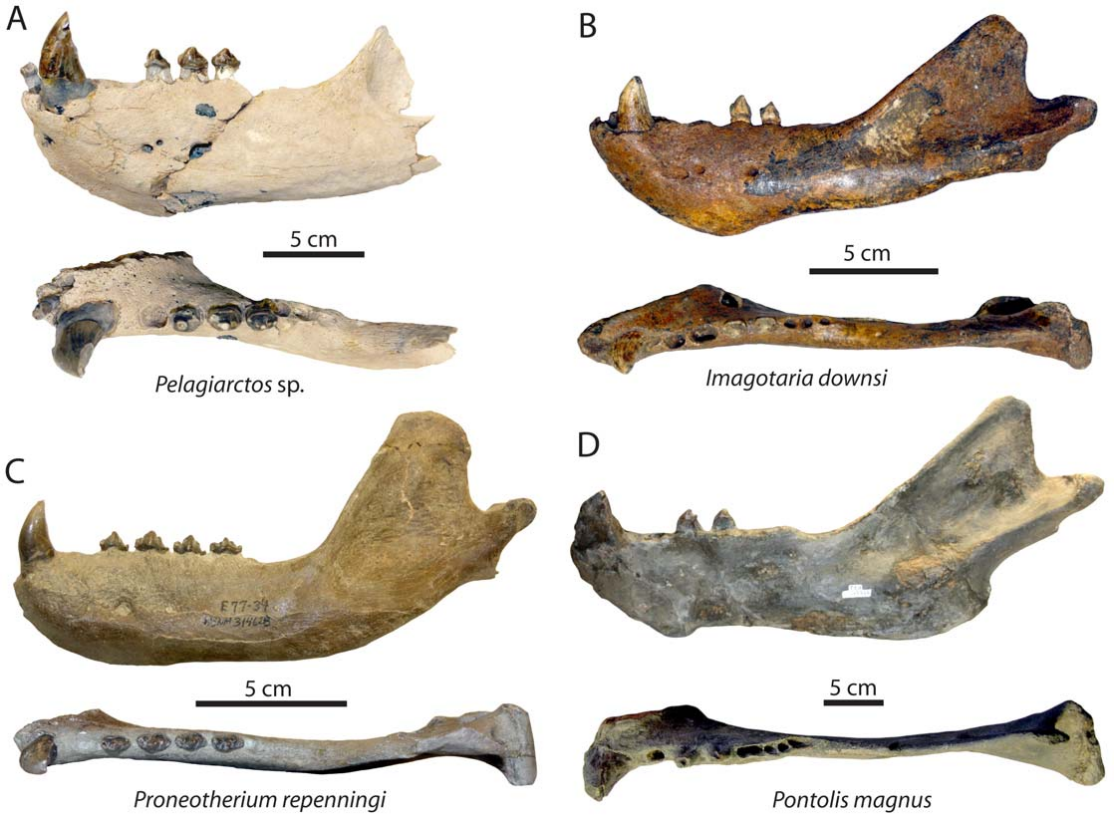
Comparison of *Pelagiarctos* sp. mandible with other Miocene “imagotariine” odobenids. A) Mandible of *Pelagiarctos* sp. (SDNHM 131041) in lateral (top) and dorsal (bottom) aspect; “Topanga” Formation, Orange County, California. B) Mandible of *Imagotaria downsi* (USNM 23858) in lateral (top) and dorsal (bottom) aspect; reflected image of right mandible; Santa Margarita Sandstone, Santa Cruz County, California. C) Mandible of *Proneotherium repenningi* (USNM 314628) in lateral (top) and dorsal (bottom) aspect; Astoria Formation, Lincoln County, Oregon. D) Mandible of *Pontolis magnus* (USNM 335563), in lateral (top) and dorsal (bottom) aspect; reflected image of right mandible; Empire Formation, Coos County, Oregon. Scale bars equal 5 cm.

Among modern and fossil pinnipeds, SDNHM 131041 shares the most similarities with early diverging odobenids (*sensu* [2]) in retaining a large and caniniform canine, an M_2_, metaconid cusps on P_2–4_, double-rooted postcanine teeth, and two lower incisors (Fig. 7). In particular, the robustness of the mandible, canines with strongly developed longitudinal sulci, bulbous postcanine tooth crowns, and presence of crenulated lingual cingulum in SDNHM 131041 are most similar to *Pelagiarctos thomasi* and *Imagotaria downsi* (Fig. 2, 7). The combination of morphological characteristics preserved in SDNHM 131041 indicates it is most similar to the fused holotype mandibles and referred postcanine teeth described for *Pelagiarctos thomasi* [19].

A few minor differences exist between *Pelagiarctos thomasi* from the Sharktooth Hill Bonebed and the new specimen (SDNHM 131041) from the “Topanga” Formation (Fig. 2). All postcanine teeth from the Sharktooth Hill Bonebed show a more strongly developed labial cingulum, and SDNHM 131041 lacks a fused mandibular symphysis, which characterizes the holotype of *Pelagiarctos thomasi*, as well as *Dusignathus seftoni*, *Valenictus*, and *Odobenus* (Fig. 2B). Because of these minor differences, SDNHM 131041 cannot be referred to *Pelagiarctos thomasi*. Although symphyseal fusion is not typically known to be variable within individual pinnipedimorph species and in this case may be a useful diagnostic criterion, we refrain from naming a new taxon due to the incompleteness of this specimen and lack of other differing characteristics, and identify SDNHM 131041 as *Pelagiarctos* sp. Ray (*in* [2]) reported that adult female *Odobenus rosmarus* occasionally retain an unfused mandibular symphysis, indicating that there is some degree of variation within the modern relative of *Pelagiarctos*; thus it is unclear whether or not this feature could be just as variable within *Pelagiarctos*. The combined possession of a fully erupted dentition, large wear facets, and large size suggest that SDNHM 131041 was an adult. The apparent maturity of this specimen thus suggests that symphyseal fusion is not an ontogenetic feature which had not yet developed (i.e. prior to the death of SDNHM 131041). Due to the small sample size, it is unclear whether the symphyseal fusion of the holotype specimen of *Pelagiarctos thomasi* is characteristic of that species, is variable, or represents a pathologic condition; further study of symphyseal fusion variability within *Odobenus* and other pinnipeds is required for further evaluation. *Pelagiarctos* sp. shares with all fossil and modern odobenids a mandible that is dorsoventrally deepest anteriorly, and differs from “enaliarctines” which are characterized by having a mandible deepest near the M_1_. Rugose and vascularized bone at the mandibular terminus distinguishes *Pelagiarctos* from *Imagotaria* and all other earlier diverging odobenids, *Dusignathus santacruzensis*, *Aivukus*, and *Protodobenus*.

A well-developed and knob-like genial tuberosity further distinguishes *Pelagiarctos* from “enaliarctines”, early diverging odobenids (*Proneotherium*, *Neotherium*), *Gomphotaria*, *Protodobenus*, and *Odobenus*. *Pelagiarctos* differs from dusignathines and odobenines in lacking an elongate mandibular symphysis and divergent mandibular rami. *Pelagiarctos* lacks an edentulous mandibular terminus, which is present in the Odobenini; furthermore, a dorsal longitudinal symphyseal furrow is also absent, unlike *Valenictus* and *Odobenus*. The horizontal symphyseal portion of the mandibular ramus in *Pelagiarctos* differs from the condition in *Ontocetus* and *Valenictus*, where it is upturned. *Pelagiarctos* exhibits a straight ventral margin of the mandible, unlike the sinuous condition in *Dusignathus*, *Pontolis*, and some specimens of *Imagotaria*.

The dentition of *Pelagiarctos* is generally plesiomorphic and close to the morphology of other early diverging odobenids (Fig. 7). For example, it differs from dusignathines, odobenines, *Pontolis*, and *Imagotaria* in the retention of a metaconid cusp and thick postcanine enamel. The presence of paraconid and hypoconid cusps, an M_2_, and double-rooted P_2–4_ further distinguishes *Pelagiarctos* from dusignathines and odobenines. *Pelagiarctos* differs from the dusignathines in retaining a vertically oriented lower canine with posterior crista and lacks longitudinal fluting. The odobenines differ from *Pelagiarctos* in exhibiting a premolariform lower canine, retaining an M_1_, lacking tooth enamel, and lacking a posterolingual shelf; *Pelagiarctos* further differs from *Odobenus* and *Valenictus* in retaining lower incisors and a P_4_. *Pelagiarctos* is distinguished from earlier diverging odobenids and “enaliarctines” in possessing several derived features. *Pelagiarctos* exhibits a short postcanine toothrow, unlike *Enaliarctos*; *Pelagiarctos* is further distinguished from *Enaliarctos*, *Pteronarctos*, and *Proneotherium* in having a reduced metaconid and an expanded talonid or posterolingual shelf. *Pelagiarctos* differs from “enaliarctines”, *Proneotherium*, *Neotherium*, and *Kamtschatarctos* in exhibiting inflated, bulbous postcanine crowns. *Pelagiarctos* sp. differs from *Proneotherium* in lacking a bicuspidate paraconid on the P_3–4_, and lacking a deep notch between the paraconid and protoconid cusps (Figure 7); the latter feature also characterizes *Enaliarctos emlongi* and *Enaliarctos barnesi* [23], but not *Pteronarctos* [39].

### Phylogenetic Analysis

**Figure 8:**
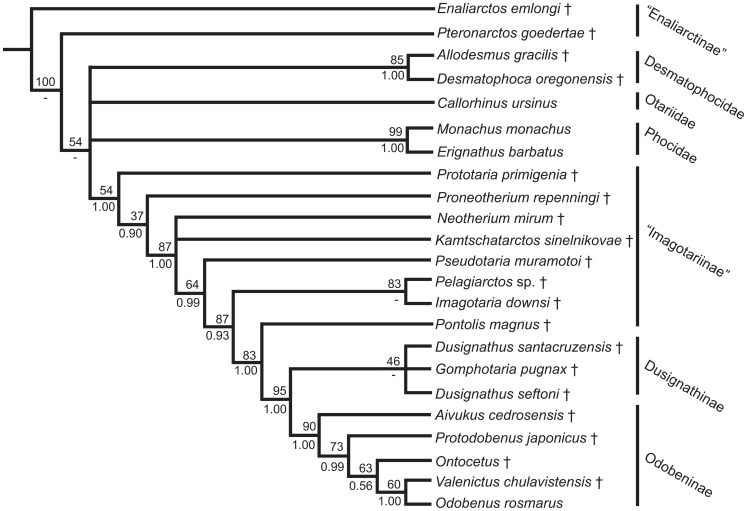
Strict consensus tree of odobenid relationships. Bootstrap support and Bayesian posterior probabilities are labeled adjacent to nodes (above and below, respectively). Odobenidae and subfamilies and tribes within Odobenidae are labeled to the right. Extinct taxa are labeled with ‘†’.

Phylogenetic analysis of the complete character set using implied weighting (K = 3) recovers one most parsimonious tree (tree length 48.31, CI = 0.564, RI = 0.730), which is shown in Figure 8. Application of different weights (K = 2–5) produced no changes in topology. When no weighting scheme was applied, the resolution of the tree is reduced, with members of Dusignathinae recovered in an unresolved polytomy with Odobeninae, and *Pelagiarctos* sp., *Imagotaria, Neotherium*, and *Kamtschatarctos* occurred in an unresolved polytomy with a clade consisting of *Pontolis*, Dusignathinae, and Odobeninae. Bayesian analysis of the character set produced a similar topology, with the main differences including the recovery of a monophyletic Phocoidea (Desmatophocidae+Phocidae) with moderate support, which in turn was sister to Otariidae; *Pelagiarctos* as the sister taxon to a clade comprised of *Imagotaria*, *Pontolis*, and all later diverging odobenids; and members of *Dusignathus* and *Gomphotaria* in an unresolved polytomy with a monophyletic Odobeninae.

The “enaliarctines” *Enaliarctos* and *Pteronarctos* are recovered as the earliest diverging taxa within this study, with *Pteronarctos* sister to crown Pinnipedia with strong support (BS = 100). However, resolution within crown Pinnipedia remains poor, although the monophyly of Desmatophocidae (BS = 85, PP = 1.00) and Phocidae (BS = 99, PP = 1.00) is recovered with strong support. Otariidae, Desmatophocidae, and Phocidae are recovered as unresolved within crown Pinnipedia.

Within Odobenidae (BS = 54, PP = 1.00), *Prototaria* is recovered as the earliest diverging walrus, the sister taxon to a poorly supported (BS = 37, PP = 0.90) clade comprised of the remainder of Odobenidae. Odobenidae is characterized by five unequivocal synapomorphies: anterior narial opening large, thick-margined and dorsoventrally elliptical (character 2); a dorsoventrally thick and laterally broad pterygoid strut (character 13); possession of a large epitympanic recess (character 23); Bony tentorium appressed to petrosal (character 29); and a triple-rooted M^1^ (character 77); One equivocal synapomorphy was also suggested, presence of well developed cuspules on the P^1–2^ cingulum (character 71).

*Proneotherium* was recovered as the next diverging lineage of walrus, the sister taxa to a poorly resolved clade comprised of all later diverging walruses. This clade is supported by one unequivocal synapomorphy, a short and slender zygomatic process of the squamosal (character 19) and one equivocal synapomorphy, absence of the fossa muscularis on the orbital wall (character 14). *Neotherium* and *Kamtschatarctos* are found in an unresolved polytomy with a clade comprised of all later diverging walruses. This clade is supported by the following six synapomorphies: ascending process of maxilla along nasal short with no contact between ascending process and frontal (character 3); palatine long and posterolaterally expanded (character 11); a narrow and parallel-sided interorbital bar (character 17); a flattened and plate-like paroccipital process (character 34); presence of a talonid basin in the form of a slight concavity or shelf in the lower postcanine dentition (character 69); and a reduced metaconid (character 70). Five equivocal synapomorphies included absence of a supraorbital process (character 16); humerus with the medial lip of the distal trochlea greater in diameter than the distal capitulum (character 84); the distal end of the radius expanded with a large radial process (character 85); insertion of the pollicle extensor and metacarpal I forms a pit (character 86); and a scapholunar forms a well-formed pit for the magnum (character 87).

*Pseudotaria* is found as the earliest diverging lineage of a clade comprised of *Pelagiarctos, Imagotaria, Pontolis*, Dusignathinae, and Odobeninae, with moderate to strong support (BS = 64, PP = 0.99). Characters that supported this topology include the following synapomorphies: a transversely arched palate (character 9); a reduced or absent pseudosylvian sulcus (character 28); and a double-rooted M^1^. The next diverging lineage identified as a strongly supported (BS = 87, PP = 0.93) clade comprised of *Pelagiarctos* sp. and *Imagotaria downsi* and all later diverging walruses. This clade is characterized by six unequivocal synapomorphies, including presence of a shallow glenoid fossa of the squamosal (character 26); a broad and pentagonal basioccipital (character 30); a large mastoid process of the squamosal (character 33); lateral lower incisors greater in size than medial incisors (character 54); bulbous postcanine crowns (character 63); and single and bilobed P^3^ roots (character 74).

Within this clade, *Pelagiarctos* sp. and *Imagotaria downsi* are recovered as sister taxa with strong support (BS = 83). Unequivocal synapomorphies for this clade included lower canine roots bilobate in cross-section (character 59) and presence of rough or crenulated postcanine lingual cingulum (character 67).

The next diverging lineage is *Pontolis*, which is recovered with strong support (BS = 83, PP = 1.00) as the sister taxon to a Dusignathinae+Odobeninae clade. Unequivocal synapomorphies for this clade included termination of mandibular symphysis at the level of P_2_ (character 42); a sinuous ventral border of the mandible (character 45); an enlarged digastric insertion (character 46); mandibular condyle elevated above the tooth row (character 47); absence of posterior crista on C_1_ (character 57); presence of thin or patchy enamel (character 62); and absence of lower postcanine paraconid cusps (character 66); and single-rooted P^4^ roots (character 76).

Dusignathinae and Odobeninae are recovered as a clade, with strong support (BS = 95, PP = 1.00). Unequivocal synapomorphies for this clade included a very broad and short interorbital bar (character 17); presence of two upper incisors (character 51); absence of hypoconid cusps on lower premolars (character 68); cingulum on P^1–2^ weak and bulbous (character 71); single-rooted P_2_ (character 72) and P_3–4_ (character 73); single and cylindrical P^3^ roots (character 74); reduced or absent P^4^ protocone shelf (character 75); single-rooted M^1^ (character 77) and M_1_ (character 79); and absence of M_2_ (character 81). Equivocal synapomorphies included a long mandibular symphysis (character 36) and a sharply divergent mandibular arch (character 44).

Dusignathinae is also recovered as monophyletic, but with poor support (BS = 46). Unequivocal synapomorphies for Dusignathinae included tusk-like upper canines without globular osteodentine (character 55); procumbency of lower canines (character 60); and presence of a single-rooted M_1_ (character 37). One equivocal synapomorphy is also found, a single-rooted M^2^ (character 80).

Odobeninae is recovered as monophyletic with strong support (BS = 90, PP = 1.00). Unequivocal synapomorphies for Odobeninae included: a very short ascending process of the maxilla (character 3); a dorsally projected postorbital process of the jugal (character 18); a reduced squamosal fossa on the external auditory meatus (character 25); premolariform I^3^ (character 52); absence of enamel in adults (character 62); and absence of M^2^ (character 80). Within Odobeninae, *Aivukus* is the sister taxon to a moderately supported (BS = 73, PP = 0.99) clade comprised of *Protodobenus* and Odobenini. Unequivocal synapomorphies that supported this topology included a palate that is arched transversely and longitudinally (character 9); tusk-like upper canines with globular osteodentine (character 55); C_1_ reduced in size and premolariform (character 56); and absence of M_1_ (character 78). Equivocal synapomorphies included a telescoped palatine that underlies the alisphenoid (character 11); a large and broad hamular process of the pterygoid (character 12); a sharply divergent mandibular arch (character 44); a mandibular condyle well elevated above the tooth row (character 47); root lobes of postcanines equal or narrower in width than the crowns (character 64); and absence of a talonid basin (character 69).

Odobenini is recovered as monophyletic but poorly supported (BS = 63, PP = 0.56). Unequivocal synapomorphies for Odobenini included: a symphyseal region composed of rugose, vascular bone (character 38) and a mandibular terminus which possesses an edentulous pad (character 41). Equivocal synapomorphies included presence of a posteriorly positioned orbital vacuity (character 20); a funnel-shaped optic foramen and orbitosphenoid (character 21); a flattened anterodorsally projected lambdoidal crest (character 32); very large mastoid processes (character 33); presence of an upturned horizontal mandibular ramus (character 39) and presence of the deltoid tubercle of the humerus on the lateral edge of the crest (character 83). Within Odobenini, *Valenictus* and *Odobenus* are recovered as sister taxa, with moderate to strong support (BS = 60, PP = 1.00). Unequivocal synapomorphies for this clade included: fusion of the mandibular symphysis (character 35); genial tuberosity developed as a small tubercle or process (character 37); presence of a longitudinal furrow on the anterior portion of the mandible (character 40); loss of lower incisors (character 53); absence of P^4^ (character 76); and M^1^ (character 77); and deltoid tubercle of the humerus located off crest (character 83).

## Discussion

### The Phylogenetic Relationships of *Pelagiarctos*

The phylogenetic analysis performed in this study is the first to include *Pelagiarctos,* and confirms that this taxon is a stem “imagotariine” walrus, in agreement with Barnes [19]. “Imagotariine” walruses are also found to be a paraphyletic assemblage of early-diverging stem taxa, a finding consistent with prior phylogenetic analyses of Odobenidae [2], [4], [5], [17]. Monophyly of Odobeninae is confirmed, with the phylogenetic relationships within this clade consistent with prior studies [2], [5].

A sister relationship between *Imagotaria* and *Pelagiarctos* is identified with excellent support on the basis of two synapomorphies. These synapomorphies included possession of lower canines bilobate in cross-section and presence of rough or crenulated postcanine lingual cingulum. Neither feature is found in other odobenids, although ontogenetic age and tooth wear may have impacted the coding of the latter character.

Our analysis identified *Pontolis* as a stem walrus sister to a clade composed of Dusignathinae and Odobeninae. Identification of *Pontolis* as a stem “imagotariine” is consistent with phylogenetic analyses of Kohno [5], [17], although the latter study found *Pontolis* to be the sister taxon to *Imagotaria*. Characters in Kohno [5] which were used support a sister taxa relationship included absence of a ventral tuberosity on the zygomatic root, and a sinuous ventral margin of the mandible. Our study interprets the absence of a ventral tuberosity as instead a character that was lost in later stem “imagotariines” and regained in the clade comprising Dusignathinae and Odobeninae. Our study also confirms the possession of a sinuous ventral margin of the mandible in *Pontolis*, but this feature is also possessed by *Dusignathus* and *Protodobenus*. This character was originally found to unite *Dusignathus* and *Pontolis* within the Dusignathinae [2]. The character is also found to be variable within *Imagotaria*, with both character states represented in the taxon.

The recovery of *Pontolis* as a stem walrus is contrary to the phylogenetic analysis presented by Deméré [2], which placed *Pontolis* as the earliest diverging lineage of a dusignathine clade. The latter study recognizes the *Pontolis* as a dusignathine walrus on the basis of one character, development of tusk-like lower canines, and three potential equivocal cranial synapomorphies. We agree that *Pontolis* possesses enlarged lower canines, but can find no other characters of the mandible or dentition that support inclusion of this genus within or as sister taxon to the Dusignathinae. Given the poor support found for Dusignathinae as well as the possibly non-monophyly of *Dusignathus*, further research on odobenid phylogeny is needed.

*Kamtschatarctos* has previously only been included within a phylogenetic analysis by Kohno [17]. *Kamtschatarctos* was recovered as a stem “imagotariine” walrus (Fig. 8), although the placement of this species within basal walruses still remains uncertain; it has been generally thought to have been an “imagotariine” walrus [4]–[6], [9], [17], [40]. Bayesian likelihood and maximum parsimony analysis supported placement of this taxon as close to *Neotherium*, and within a grade of stem walruses intermediate between *Proneotherium* and *Pelagiarctos-Imagotaria*. *Kamtschatarctos* is a fragmentary taxon [41] with little re-examination since its description, and we were only able to examine casts and photographs of the holotype jaw. Re-examination of the holotype and incorporation of additional cranial and postcranial characters may improve the resolution of this taxon within Odobenidae.

### Odobenid Dental and Mandibular Evolution

Walruses have long been recognized as being more diverse in the geologic past both in terms of numerical richness and morphology [2], [7], [19]. While the earliest walruses closely resembled “enaliarctine” pinnipedimorphs in overall morphology, by the latest Miocene and Pliocene Dusignathine and Odobenine walruses had diversified into a number of derived morphologies. Among these were strange double-tusked suction feeding walruses (*Dusignathus* and *Gomphotaria*, [42]), tuskless and possibly piscivorous odobenines (*Aivukus* and *Protodobenus*; [40]), and tusked odobenine walruses which were dedicated suction-feeding molluskivores (*Ontocetus*, *Valenictus*, and *Odobenus*; [1]–[3], [40], [42]). The ecological diversity of walruses in the geologic past is reflected in the diverse dental and cranial modifications of these various fossil walruses. Previous authors have summarized changes in cranial morphology and the dentition [2], [42], and we provide a supplementary discussion of the evolution of the mandible and lower dentition in odobenids.

**Figure 9:**
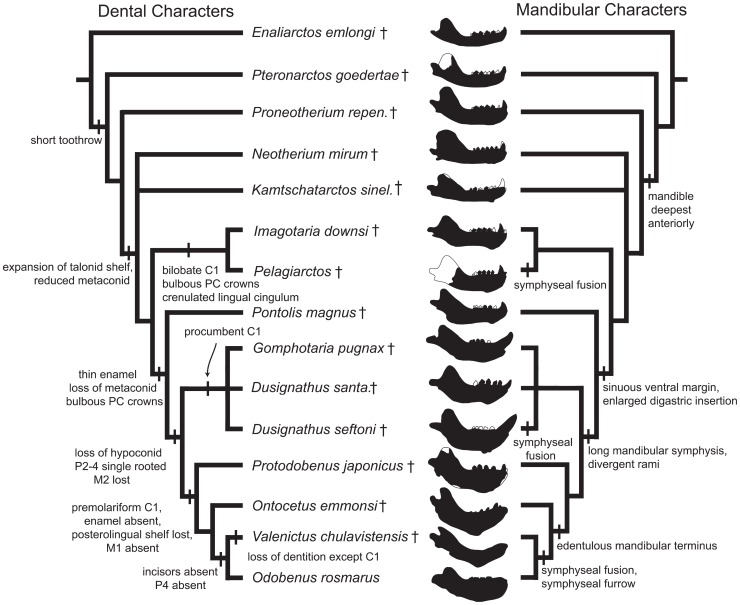
Hypothesized sequence of mandibular and dental character transformations during odobenid evolution. Dental characters shown on left cladogram, and mandibular characters shown on right cladogram, with diagrams of mandibles adjacent to taxon names; white indicates unknown morphology. Character acquisition and loss mapped directly from results of cladistic analysis, with the exception of “short tooth row”, which was mapped *a posteriori*.

*Pelagiarctos* is distinctive in being the earliest known pinniped to evolve symphyseal fusion, a condition which is limited to the odobenids (among pinnipedimorphs). Examination of this character in a phylogenetic context suggested that symphyseal fusion evolved three times (Fig. 9): once in *Pelagiarctos thomasi* [19], once in *Dusignathus seftoni* [3], and once in the common ancestor of *Valenictus* and *Odobenus* [2], [3].

In lateral view, some “enaliarctine” mandibles (*Enaliarctos emlongi*, *Pteronarctos goedertae*) are dorsoventrally deepest posteriorly near the molars, reflecting the primitive carnivoran condition where the mandible is thickest near the large carnassials, and becomes shallower nearer the anterior dentition ([23], fig. 7a therein). *Pelagiarctos*, and all other odobenids (and most otariids) instead possess a mandible that is dorsoventrally deepest anteriorly near the canines and symphysis (Fig. 7, 9). *Enaliarctos, Pteronarctos*, and the possible early pinnipedimorph *Puijila* retain the primitive ‘fissiped’ condition where the middle of the mandible is most robust adjacent to the carnassials [23], [43]. The carnassial morphology was lost early in pinniped evolution during the transition from chewing and shearing to pierce feeding [42]; additionally, pinnipeds possess larger lower canines and robust anterior portions of the mandibles relative to terrestrial carnivorans. Early diverging walruses such as *Proneotherium* and *Neotherium* have completely lost carnassial-like postcanines [4] and have an anteriorly deep mandible (Fig. 9).

Other mandibular changes occurred within the Dusignathinae and the Odobeninae. Dusignathines are characterized by a sinuous lower margin of the mandible, owing to the posteriorly enlarged digastric insertion; it is unclear whether this is related to stronger gular muscles associated with suction feeding. A posteriorly enlarged digastric insertion is also present in some phocoids (e.g. *Allodesmus*, *Acrophoca*, *Erignathus*, *Mirounga, Piscophoca*) and an otariid (*Otaria*). Odobenine mandibles show further modifications from the ancestral pinniped condition, including a mandibular symphysis that is greatly elongated and widely flaring mandibular rami, the latter relating to the extreme shortening of the rostrum [2]. The anterior part of the horizontal ramus is dorsally upturned within *Ontocetus* and *Valenictus* (Fig. 9), and in *Valenictus* and *Odobenus*, the mandibular terminus exhibits a dorsal furrow and forms a small, edentulous mandibular “pad”. Extant *Odobenus rosmarus* and the extinct *Odobenus mandanoensis* share a hypertrophied and pachyostotic symphyseal region that is transversely thickest ventrally and narrower dorsally, whereas the primitive condition possessed by all other pinnipeds is a mandible that is widest dorsally [2]; rostral and cranial pachyostosis [44] possibly functions as ballast during benthic foraging.

Changes in the dentition of odobenids can generally be placed into five categories: 1) postcanine crown simplification, 2) tooth root fusion, 3) tooth reduction, 4) changes in tooth placement, and 5) tusk development. The postcanine dentition of extant walruses is remarkably simple in comparison to earlier diverging taxa like *Neotherium* and *Pelagiarctos*. Simplification of postcanine crowns was achieved by stepwise loss of the paraconid, metaconid, and hypoconid cusps (Fig. 9); these are all present (in addition to the protoconid or principal cusp) in *Proneotherium*, *Neotherium*, and *Pelagiarctos*. The metaconid was lost in *Imagotaria*, and the paraconid and hypoconid cusps disappeared within the dusignathines and odobenines, resulting in a simple, peg-like crown (Fig. 9).

Fusion of tooth roots was a major dental transition within the Odobenidae [2], [7], and the early diverging walrus *Imagotaria downsi* was noted to have a stage of intermediate root fusion where the anterior postcanines were single-rooted or fused (but bilobate), and the posterior postcanines retained double-roots [7]. *Pontolis* exhibits a similar (but slightly more derived) pattern; dusignathines and all odobenines possess single-rooted postcanine teeth [2], [7], [40]. This same pattern of root fusion, beginning with fusion of the anterior premolar roots and progressing posteriorly, also occurs during the evolution of otariids [45], [46]. Adam and Berta [42] suggested that the anterior migration of the M_1_ in early pinnipedimorphs reflects an adaptation towards increasing functional gape. Because double-rooted teeth are anteroposteriorly more elongate than single-rooted teeth, Boessenecker [46] further argued that fusion of postcanine tooth roots was also an adaptation for increasing functional gape by crowding teeth anteriorly. Such a transition has occurred at least eight times, including twice within Otariidae (*Callorhinus,* as well as the clade composed of “Otariinae”+southern fur seals), twice within Odobenidae, once within Desmatophocidae, and three times within Phocidae (*Mirounga, Cystophora*, and *Halichoerus*; using the phylogeny of Fulton and Strobeck [15]; modified from Boessenecker [46]). The existence of unpublished latest Miocene specimens of *Imagotaria* with single-rooted teeth [2] and an unpublished early late Miocene *Gomphotaria* with some double-rooted teeth [47] suggests that root fusion may have occurred three times within odobenids (once in dusignathines, once in odobenines, and once in *Imagotaria*, totaling nine times within pinnipeds).

Reduction of teeth began with geochronologically late “imagotariines” like *Imagotaria*, with the incipient loss of the M_2_; although the M_2_ is present in *Pontolis* [2], it is present in some specimens of *Imagotaria downsi* and absent in others [7], [48]. The lower incisors were reduced and lost in dusignathines, with one incisor present in *Dusignathus seftoni*, and none in *Gomphotaria*; incisor loss independently occurred in the common ancestor of *Odobenus* and *Valenictus*. Incisor loss in odobenines may be related to allowing an oral pathway for suction feeding [42], whereas in *Gomphotaria*, it is probably related to lower canine enlargement. Further postcanine reduction occurred in odobenines with the loss of the M_1_ in *Ontocetus* and *Protodobenus*, and further loss of the P_4_ in *Odobenus*. Also within odobenines, the C_1_ is reduced and premolariform. Extreme tooth reduction characterizes the most derived walrus, *Valenictus chulavistensis*, which lost all teeth with the exception of the C^1^ [3]. Further tooth reduction within odobenines and dusignathines includes thinning and complete loss of tooth enamel [2], a feature which has also evolved within several cetacean lineages [49]–[51] and may reflect reduced reliance on dentition in the processing of prey.

Numerous changes occurred regarding the placement of anterior teeth, probably as a result of upper canine enlargement. The lateral upper and lower incisors (I^3^ and I_3_) are enlarged in many pinnipeds, and the medial incisors are often smaller and within odobenids are the first to be lost [2]. Decreased size of the medial incisor (I_2_) in early diverging odobenids is also associated with it being positioned posteromedial to the lateral incisor, so that the canines and incisors do not form a transverse arc, but instead are medially indented; this condition is present in *Pelagiarctos*, *Neotherium*, *Imagotaria*, and *Ontocetus*, and also many otariids and phocids [26].

Tusk development occurs within the dusignathine and odobenine walruses, with the development of modest upper and lower tusks (Dusignathinae) and the development of very elongate upper tusks (Odobenini). Within Dusignathinae, the tusks of *Dusignathus seftoni* and *Gomphotaria* are not only greatly enlarged, but also procumbent ([1], [3]; Fig. 9). The function of upper and lower tusks within these taxa remains unclear. Elongation of tusks in the Odobenini was achieved by the addition of a globular dentine column within the tusks, a dental tissue that is unique to the Odobenini, and lacking in dusignathines [1]–[3]. Deméré [3] suggested that tusk evolution was driven by social and sexual display-related behavior; in modern *Odobenus* tusks are prominently displayed during aggressive confrontations [52] as well as used in intraspecific combat [53]. Muizon et al. [54] however, argued that the convergent evolution of tusks in the odontocete *Odobenocetops* suggests tusk evolution is related instead to feeding. Tusks in walruses may function as ‘sled runners’ on the seafloor, allowing the animal to orient its body with the sea floor by resting on its tusks [54]. This behavior has since been observed in the wild [55] and is also supported by wear patterns on the tusks. However, it is important to note that tusks evolved twice within odobenids, and within the dusignathines and basal odobenines, short procumbent tusks could scarcely serve such a function. Early evolution of tusks was thus likely driven by social interaction, and only later was exapted for use in feeding.

### The Feeding Ecology of *Pelagiarctos* Revisited

With the description of the new material herein, *Pelagiarctos* (Fig. 2) has now been documented from both the “Topanga” Formation and the Round Mountain Silt (Fig. 1), two formations roughly correlative in age (Howard and Barnes, 1987). Although only 320 km south of exposures of the Round Mountain Silt at Sharktooth Hill, adjustment for strike-slip offset since deposition of the “Topanga” Formation suggests the two were originally separated by 620 km [29], indicating that fossils of *Pelagiarctos* have been found along a long section of the eastern North Pacific coastline. Within the Round Mountain Silt, three genera of pinnipeds are recognized, the desmatophocid *Allodesmus* [25], [56], [57], the early “imagotariine” *Neotherium* [17], [56], and *Pelagiarctos* [19]. Two other taxa may also be present within the Round Mountain Silt (Desmatophocine “A” and Desmatophocine “B”), although they are poorly known [58]. The “Topanga” Formation pinniped assemblage remains poorly known; however, material referable to *Allodesmus*, *Neotherium*, and an undescribed pinniped have been noted as occurring within the fauna [29].

*Allodesmus* is the most abundant pinniped taxon represented within the Round Mountain Silt, with possibly one to three species represented [25], [58], [59]. *Allodesmus* was a large pinniped with enlarged orbits, simplified dentition, and strong sexual dimorphism [57]. These traits are found in modern elephant seals (*Mirounga*; [57]), and may suggest that *Allodesmus* was a deep-diving pelagic pinniped [60]. The second most common pinniped taxon is *Neotherium mirum*, a small walrus lacking many of the specialized suction-feeding adaptations of later walruses, and has been previously interpreted as a piscivorous generalist feeder [17]. Barnes [19] suggested that niche partitioning or inhabitation of this region in different seasons may have reduced competition and fostered such a diverse pinniped assemblage.

Although known only from mandibles and isolated teeth, Barnes [19] suggested that the unique morphology preserved in both the holotype and referred material of *Pelagiarctos* indicated occupation of a different niche than contemporary pinnipeds. Barnes [19] proposed a novel interpretation of the feeding ecology of *Pelagiarctos thomasi* as a macrophagous predator, based on four lines of evidence: 1) The robust morphology of the mandible; 2) the perceived similarity of the postcanine teeth with scavenging carnivorans (extant hyaenids and extinct borophagine canids); 3) The inferred large body size of *Pelagiarctos*; and 4) the apparent rarity of *Pelagiarctos* within the Sharktooth Hill Bonebed.

The mandible of *Pelagiarctos* is far larger and more robust than that of *Neotherium*, and the holotype mandible possesses a large and fused bony symphysis, unlike other contemporary pinnipeds. Symphyseal fusion is not present in the “Topanga” Formation specimen, although the mandible is similarly robust. Fusion and ankylosis of the mandibular symphysis, among pinnipeds, is only typically found in *Pelagiarctos thomasi*, *Dusignathus seftoni*, and the odobenines *Odobenus* and *Valenictus*; in odobenines it is hypothesized to reduce torsion during suction feeding [42]. Among terrestrial carnivores, mandibular symphyseal fusion occurs in a variety of taxa, including big cats (*Panthera*), aardwolves (*Proteles*), bears (Ursidae), kinkajou (*Potos*) and a variety of mustelid taxa [61]. Within terrestrial taxa, this feature is thought to reduce strain at the symphyseal joint and focus force along the horizontal midline of the mandibles [62]. Within the Feliformia symphyseal fusion is strongly correlated with taxa that regularly consume large struggling prey, however diet and symphyseal fusion appear to be only poorly correlated in caniforms [61]. Barnes [19] argued that symphyseal fusion might be related to a durophagous diet and an adaptation to processing the bones of large prey. However, most durophagous taxa such as borophagine canids, hyenas, and sea otters have loose, unfused mandibular symphyses, which may relate to the need to rotate the jaws when applying force to particularly tough foods such as shellfish or bone, to reduce tooth breakage [62]. Fusion of the mandibular symphysis would have probably increased tooth breakage if *Pelagiarctos* had a durophagous diet, something not observed in the new specimen or the previously described material. Given the poor correlation between diet and symphyseal fusion between caniforms, use of this character to determine the ecology of *Pelagiarctos* is probably premature, and cannot be used as evidence for hypercarnivory.

The dentition of both specimens of *Pelagiarctos* is similar to that of *Neotherium* and other earlier diverging “imagotariines”, with a prominent protoconid and less prominent metaconid and paraconid cusps (Fig. 5–6). Simplification of dentition as seen in later diverging suction-feeding odobenids (Dusignathinae and Odobeninae [42]) is not present in *Pelagiarctos* sp. The number of incisors has not been reduced as seen in later diverging odobenids, a feature that may be related to accommodating the movement of food while the mouth is closed during suction feeding. Most of the characters of the dentition associated with a macropredatory lifestyle cannot be identified within the holotype or referred material of *Pelagiarctos*, including enlarged procumbent incisors and canines [42], [63], sharp postcanine cusps (*Hydrurga*; [42], [63], and presence of carinae on the anterior dentition [63]. Prominent anterior wear facets are present, as seen in taxa such as *Dusignathus santacruzensis* and *Pontolis magnus*, and imply that the teeth are still used in the capture and processing of prey. This is in contrast to the wear observed in later odobenines such as *Odobenus*, in which the teeth are polished as a result of inhalation of grit and movements of the tongue [64].

Overall, the morphology of *Pelagiarctos* suggests that *Pelagiarctos* was probably not a specialized suction feeder, given the distinct wear facets, complex dentition, and retention of incisors. A durophagous diet is also unlikely. Barnes [19] suggested that *Pelagiarctos* may have been “an active marine predator with strong crushing and biting abilities” ([19]:9), based on superficial similarities of the cheek teeth (i.e. shape, size, and position on the mandible) with hyenas and borophagine canids, as well as fusion of the mandibular symphysis. We do not find any convincing evidence of this in the dentition of *Pelagiarctos*, as there is little overall similarity between its teeth and borophagine canids, or hyaenids. Although the cheek teeth of *Pelagiarctos* are large (in terms of absolute size), they are small relative to the size of the mandible. *Pelagiarctos* postcanine teeth are superficially similar to anterior premolars of borophagines and hyaenids, and although Barnes [19] noted sharp anterior and posterior cristae on these teeth, we were unable to identify any such cristae on teeth referred to *Pelagiarctos thomasi* by Barnes [19]; instead, they appear to be rather blunt. *Pelagiarctos* generally lacks sharp cusps or cristae of any sort, unlike the aforementioned borophagous fissipeds. In general, the dentition of *Pelagiarctos* is similar to other early odobenids which have been interpreted as generalist piscivores.

**Figure 10:**
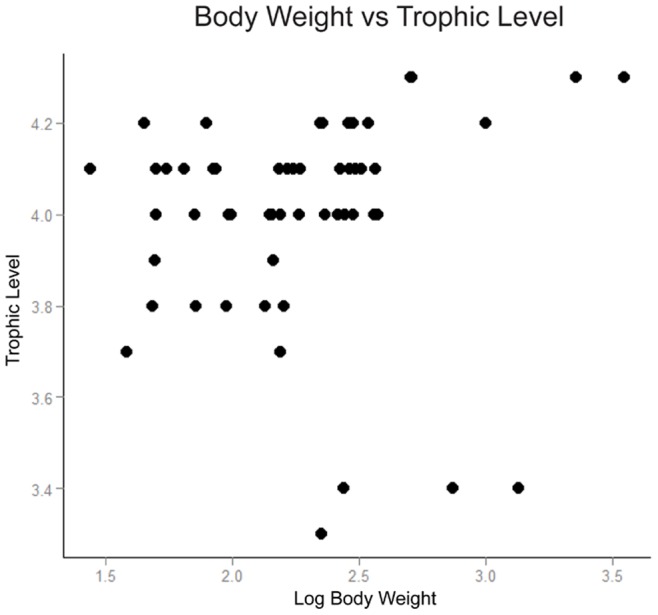
Relationship between body weight and calculated trophic level among modern pinnipeds. Taxa with strong sexual dimorphism are represented by separate points for male and female taxa. Note that the greatest body weight is associated with the highest and the lowest trophic levels, and the lack of a clear trophic level trend for lower body weights.

The body size of *Pelagiarctos* may also provide useful information on its ecology. Based on length of the lower tooth row of SDNHM 131041 and linear regression equations based on measurements of modern taxa (Churchill unpublished data), we estimate that *Pelagiarctos* weighed ∼350 kg, about the size of an adult male South American Sea Lion (*Otaria*). *Pelagiarcto*s was the first truly large odobenid, and started a trend towards ever larger body sizes within later diverging odobenids. The contemporary pinnipeds *Neotherium* and *Allodesmus* would have had weighed ∼280 and up to 1400 kg respectively, making *Pelagiarctos* intermediate in body size between the two taxa. Large body size has been indicated to be correlated with trophic level [65], [66], with larger carnivores being able to feed on larger size classes of prey. To test whether body size relates to trophic level for pinnipeds, we plotted log body weight data from Lindenfors [67] versus trophic level data from Pauly et al. [68]. Trophic level data for pinnipeds is based on categorization of pinniped diet based on stomach contents and behavioral and morphological data. All taxa with strong sexual dimorphism are treated separately, but with the same trophic level. No correlation is found between log body weight and trophic level (α = 0.05, p = 0.554, adjusted R^2^ = −0.01211; GLM analysis performed in R 2.15; Fig. 10). When comparing trophic level and body size, the largest pinnipeds are found to occupy the highest trophic levels (e.g. *Mirounga*) and the lowest trophic levels (e.g. *Odobenus* and *Erignathus*; Fig. 10). In part this is because pinnipeds are generalist feeders; even taxa with fairly derived morphologies are likely to feed on a range of prey items. A significant component of the diet of the “macropredatory” *Hydrurga* is krill [69], while the specialized molluskivore *Odobenus* has been documented to kill and eat other seals [70]. The estimated body size of *Pelagiarctos* places this taxon within range of body sizes reported for generalist feeders, suggesting that this taxon did not occupy a low trophic level (as reported for the much larger *Odobenus* and other molluskivores) nor within the range of body size reported for the high trophic level *Mirounga*.

The rarity of *Pelagiarctos* within the fossil record has also been cited as a possible indicator of its ecology [19]. Although the Sharktooth Hill Bonebed, the type locality for *Pelagiarctos*, preserves one of the largest and most diverse marine vertebrate assemblages of the Neogene and has been prospected for decades, remains of *Pelagiarctos* from this locality remain limited to six isolated teeth and a partial mandible. Given the abundance of fossil material referable to *Neotherium* and *Allodesmus*, this rarity appears to be a real phenomenon. Barnes [19] argued that the rarity as well as size of *Pelagiarctos* may correlate with a fairly high trophic level, and that a significant portion of its diet may have consisted of other pinnipeds, limiting the population to a level far below that of contemporary pinnipeds. He identified the holotype as a male individual, based on the presence of large broken canines and a fairly rugose mandible; because some isolated cheek teeth fit into alveoli of the holotype mandible, Barnes [19] further identified these isolated teeth as representing males. He used this identification to argue that *Pelagiarctos* possessed a partially allopatric distribution of genders, with females not ranging into the Temblor Sea. Given the limited amount of material of *Pelagiarctos* available for study and scarcity of studies quantifying sexual dimorphism in modern and fossil pinnipeds, evaluation of sexual dimorphism is probably premature and we question gender identification of isolated teeth. Other explanations for the rarity of *Pelagiarctos* within the Round Mountain Silt also exist. Many seals are prone to vagrancy [71]–[74], and the rarity of an individual taxon within a specific formation may reflect only occasional occurrence within the given region. In addition, the Sharktooth Hill bonebed was formed over a protracted period of time, possibly as long as 700 ka [75]. The rarity of *Pelagiarctos* within this fossil locality may indicate the existence of brief periods of unusual climate, which in turn could have led to shifts in the species composition of the local fauna and preservation of taxa not normally present along this section of coastline.

### Conclusions

New fossil material of the enigmatic early walrus *Pelagiarctos* includes a pair of well preserved mandibles from the lower-middle Miocene “Topanga” Formation of Orange County, California. This fossil is more complete than the fragmentary holotype of *Pelagiarctos thomasi*, and confirms the referral of isolated teeth from the Sharktooth Hill Bonebed to this taxon. Because of minor differences including the lack of a fused mandibular symphysis, this new specimen is not referable to *P. thomasi* and is instead identified as *Pelagiarctos* sp. Owing to the more complete preservation of this new specimen, inclusion of *Pelagiarctos* within a phylogenetic analysis was possible for the first time, clearly establishing it as an early diverging odobenid, and as a sister taxon of *Imagotaria*. Although previously hypothesized to be a macrophagous predator, reevaluation of the evidence failed to support such an interpretation. Previously identified lines of evidence may have other explanations (rarity of *Pelagiarctos* within the Sharktooth Hill Bonebed), do not apply to pinnipeds (correlation of body size to trophic level), or were not verified in this study (borophagine/hyaenid like postcanine dentition with sharp cristae). The robust and fused mandibular symphysis may genuinely support the macrophagy hypothesis, but other evidence is instead suggestive of a generalist piscivore diet for *Pelagiarctos*.

## Supporting Information

Text S1 **Includes a list of specimens examined during this study and a list of museum abbreviations.**
(DOC)

Text S2 **Includes a list of detailed descriptions for cladistic characters used in the phylogenetic analysis.**
(DOC)
