# Supplementary material for: A Reevaluation of the Morphology, Paleoecology, and Phylogenetic Relationships of the Enigmatic Walrus Pelagiarctos
Source: PLoS One. 2013 Jan 16;8(1):e54311. doi: 10.1371/journal.pone.0054311 (PMC3546998; doi:10.1371/journal.pone.0054311)
Supplement: Text S1 — Includes a list of specimens examined during this study and a list of museum abbreviations. (DOC) [file pone.0054311.s001.doc]

**A Reevaluation of the Morphology, Paleoecology, and Phylogenetic Relationships of the Enigmatic Walrus *Pelagiarctos***

Robert W. Boessenecker^1,2^*, Morgan Churchill^3,4^

1. Department of Geology, University of Otago, 360 Leith Walk, Dunedin, New Zealand

2. Research Associate, University of California Museum of Paleontology, Berkeley, California, 94720

3. Department of Geology and Geophysics, University of Wyoming, Laramie, Wyoming, 82071

4. Program in Ecology, University of Wyoming, Laramie, Wyoming, 82071

**Supporting Information Text S1 - List of examined cranial and mandibular material.**

Abbreviations for specimens included within this study are as follows: CAS, California Academy of Science, San Francisco, California; CBM, Natural History Museum and Institute, Chiba, Japan; CM, Carnegie Museum of Natural History, Pittsburg, Pennsylvania, USA; HUMZ, Laboratory of Marine Zoology, Hokkaido University, Hakodate, Japan ; HUTE, Geoscience Institute, Hyogo Prefecture, Japan; IGCU, Instituto de Geología, Ciudad Universitaria, Universidad Nacional Autónoma de México, Mexico City, Mexico; KUZ, Kyoto University Museum, Kyoto, Japan; LACM, Natural History Museum of Los Angeles County, Los Angeles, California, USA; NSMS, National Museum of Nature and Science; PIN, Palaeontological Institute, Russian Academy of Sciences, Russia; SDNHM, San Diego Natural History Museum, San Diego, California, USA; SFM, Shinshushinmachi Fossil Museum, Miyagi, Japan; UCMP, University of California Museum of Paleontology, Berkeley, California, USA; UOMNH, University of Oregon, Museum of Natural History, Eugene, Oregon, USA; USNM, National Museum of Natural History and Culture, Smithsonian Institution, Washington D.C., USA; UWBM, Burke Museum of Natural History, University of Washington, Seattle, Washington, USA.

*Aivukus cedrosensis*

IGCU 901 (cast)

*Allodesmus gracilis*

SDNHM 116702

LACM 21092

UCMP 81708

UCMP 194003

*Callorhinus ursinus*

UWBM 18335

UWBM 18336

*Desmatophoca oregonensis*

LACM 123285

UOMNH F735 (cast)

USNM 335430

USNM 335457

USNM 335478

USNM 335723

*Dusignathus santacruzensis*

UCMP 27121

SDNHM 68981

*Dusignathus seftoni*

SDNHM 20801

SDNHM 90507

*Enaliarctos emlongi*

USNM 250345

*Erignathus barbatus*

CM 15316

KUZ 1695

HUMZ/A-15

*Gomphotaria pugnax*

LACM 121508

*Imagotaria downsi*

SBMNH 342 (cast)

USNM 83858

USNM 184060

UCMP 88459

*Kamtschatarctos sinelnikovae*

PIN 3016/10-11 (cast)

*Monachus monachus*

USNM 219059

*Neotherium mirum*

LACM 12300

LACM 131950

UCMP 81665

UCMP 110618

*Odobenus rosmarus*

CAS 261

NSMT 27861, 27862

UWBM 80659

*Ontocetus* spp.

IRSNB M.168 (cast)

SFM-CV-0001 (cast)

USNM 9343

*Pelagiarctos* sp.

SDNHM 131041

*Pelagiarctos thomasi*

LACM 121501

LACM 123415

LACM 38812

LACM 7856

UCMP 93058

*Pontolis magnus*

USNM 335563

USNM 335567

*Proneotherium repenningi*

USNM 205333

USNM 205334

USNM 215068

USNM 335526

USNM 314628

USNM 335697

USNM 314541

*Protodobenus japonicus*

NMNS-PV 22194 (cast)

*Prototaria planicephala*

SSSME 13317 (cast)

*Prototaria primigena*

HUTE 10001 (cast)

*Psuedotaria muramotoi*

CBM-PV 382

*Pteronarctos goedertae*

LACM 127972

USNM 335432

USNM 167648

*Valenictus chulavistensis*

SDNHM 36786

SDNHM 38227

SDNHM 83717

SDNHM 83719

SDNHM 90497
