# Supplementary material for: A Reevaluation of the Morphology, Paleoecology, and Phylogenetic Relationships of the Enigmatic Walrus Pelagiarctos
Source: PLoS One. 2013 Jan 16;8(1):e54311. doi: 10.1371/journal.pone.0054311 (PMC3546998; doi:10.1371/journal.pone.0054311)
Supplement: Text S2 — Includes a list of detailed descriptions for cladistic characters used in the phylogenetic analysis. (DOC) [file pone.0054311.s002.doc]

**A Reevaluation of the Morphology, Paleoecology, and Phylogenetic Relationships of the Enigmatic Walrus *Pelagiarctos***

Robert W. Boessenecker^1,2^*, Morgan Churchill^3,4^

1. Department of Geology, University of Otago, 360 Leith Walk, Dunedin, New Zealand

2. Research Associate, University of California Museum of Paleontology, Berkeley, California, 94720

3. Department of Geology and Geophysics, University of Wyoming, Laramie, Wyoming, 82071

4. Program in Ecology, University of Wyoming, Laramie, Wyoming, 82071

**Supporting Information Text S2 – Character list for phylogenetic analysis.**

1. Premaxilla: rostral process. 0= absent. 1= present.

Although absent (state 0) in *Monachus*, ursids, Desmatophocidae, and *Enaliarctos*, a prominent knob-like rostral process on the anterodorsal tip of the premaxillae (state 1) is present in many modern and fossil pinnipeds. A rostral process is present in *Odobenus* and all fossil odobenids with well-preserved rostra, Otariidae, *Pteronarctos*, *Pinnarctidion*, and *Pacificotaria*. In *Odobenus*, the rostral process is elevated along with the external nares above the ventral margin. Character 3 of Berta and Wyss , modified by Kohno ([1]: character 1).

2. Anterior narial opening. 0= large, thin margin, and rounded. 1= large, thick margin, and dorsoventrally elliptical. 2= large, thick margin with prenarial shelf.

The bony nares are primitively large and round in anterior aspect with a thin ventral margin (state 0) in pinnipedimorphs and terrestrial carnivores. In odobenids, the bony nares are transversely compressed and elliptical in anterior aspect, and separated from the alveolar margin by a thick alveolar process of the maxilla (state 1). This condition is exaggerated in the Odobenini (*Odobenus*, *Ontocetus*, and *Valenictus*) where the bony nares are elevated high above the alveolar margin [2]. *Allodesmus* exhibits large nares that are posteriorly retracted behind an anteroposteriorly elongate prenarial shelf (state 2; [3]). This condition is somewhat paralleled in *Pontolis magnus*, although coded as state 1 due to the oval-shaped bony naris. Character 2 of Kohno [1], modified from character 6 of Kohno et al. [4] and character 1 of Deméré [2].

3. Premaxilla: ascending process along nasal. 0= overlap with nasal long, little contact of nasal and maxilla. 1= short overlap, no contact of ascending premaxilla and frontal. 2= very short overlap.

In *Enaliarctos*, *Pteronarctos*, *Callorhinus*, *Prototaria*, and *Proneotherium*, the ascending process of the premaxilla is long and extends between the nasal and maxilla, with a long contact between the nasal and premaxilla so that contact between the nasal and maxilla is limited (state 0). Two derived conditions exist. In later diverging “imagotariines” (*Neotherium*, *Pseudotaria*, *Imagotaria*, *Pontolis*), *Dusignathus seftoni*, and *Gomphotaria*, the ascending process of the premaxilla has a short contact with the nasal, and the premaxilla and frontal are separated (state 1). The other derived condition is present in Desmatophocidae, Phocidae, and Odobenines, which have a very short and limited overlap between the premaxilla and nasal. Character 3 of Kohno [1], modified from character 2 of Deméré [2].

4. Nasal: posterior end. 0= converging. 1= parallel with transversely straight frontal/maxillary suture. 2= parallel with broad V-shaped frontal/maxillary suture. 3= diverging. 4= penetrating frontal.

The form of interdigitation of the rostral bones and the frontal varies among pinnipeds, and several conditions are present. In the primitive condition, the posterior margins of the nasals are convergent and terminate along a transversely oriented line formed by the frontal-maxillary suture (state 0); this condition is present in *Enaliarctos*, *Pteronarctos*, *Prototaria*, *Proneotherium*, *Neotherium*, and *Pseudotaria*. In some later diverging odobenids, the frontal/maxillary suture is straight and posteriorly convergent, forming a V-shape; the nasals terminate along this line at the posterior apex of the ‘V’ (state 1). This condition characterizes *Dusignathus seftoni*, *Gomphotaria*, *Pontolis*, crania of *Imagotaria* sp. cf. *I.* *downsi* (see Deméré, [2]: 114), as well as *Ontocetus* and *Valenictus*. In some odobenines (*Protodobenus*, *Odobenus*) the frontal-maxillary suture is straight and transversely directed, and the posterior ends of the nasals terminate along a transverse line (state 2). Deméré [2] considered this state to characterize *Imagotaria* (which in his analysis was a combined OTU including *Imagotaria downsi* and *Imagotaria* sp., cf. *I. downsi* from the late Miocene Empire Formation of Oregon), but because no specimen of *Imagotaria downsi* includes nasal bones we coded this taxon as ‘?’. Furthermore, the condition in specimens of *Imagotaria* sp., cf. *I. downsi* (USNM 335599, 335594) is a slight V-shape intermediate between states 1 and 2. In *Callorhinus* and other Otariidae, the posterior tips of the nasals diverge posteriorly and extend slightly into the frontal (state 3). The last derived character state describes the condition in Phocidae and Desmatophocidae, where the nasals extend posteriorly and deeply divide the frontals along the midline (state 4). Deméré [2] considered a V-shaped frontal-maxillary suture to be a synapomorphy of the Dusignathinae. However, Kohno [1] indicated that a Pliocene skull of *Ontocetus* from Japan exhibits a V-shaped suture, and coded *Valenictus chulavistensis* as this state as well. Our observations of *Valenictus chulavistensis* (e.g. SDNHM 83719, 90497) confirm those of Kohno [1]. In general, state 1 seems to characterize later-diverging odobenids crownward of *Imagotaria*, with a transverse suture (state 2) evolving within odobenines. Character 4 of Kohno [1], modified from characters 3 and 4 of Deméré [2].

5. Incisive foramina. 0= distinct pair. 1= foramina coalesced. 2= trilobed pits. 3= single pit or reduced.

The incisive foramina are positioned anteromedially on the palate and lie medial to the canines. They are primitively paired (state 0) in pinnipedimorphs, and this condition characterizes "enaliarctines", *Callorhinus*, Phocidae, and most odobenids. The foramina are coalesced (state 1) in *Desmatophoca*, and are present as trilobate pits (state 3) in *Allodesmus*, *Dusignathus* *seftoni*, and *Ontocetus*. In *Odobenus*, the foramina are reduced to a single pit. Character 5 of Kohno [1].

6. Infraorbital foramen. 0= small. 1= enlarged.

Although Berta and Wyss [5] considered an enlarged infraorbital foramen to be a synapomorphy of Pinnipedimorpha (in comparison to the small foramina of terrestrial arctoids), greatly enlarged foramina are present in some odobenids (*Dusignathus seftoni*, and odobenines). In contrast, all other odobenids, "enaliarctines", otariids, desmatophocids, and phocids have comparatively smaller infraorbital foramina. The infraorbital foramen transmits the infraorbital artery and infraorbital nerve, part of the maxillary branch of the trigeminal nerve. The infraorbital nerve and artery innervate and supply blood to muscles and tissues of the lips and vibrissae. An enlarged infraorbital foramen is associated with enlarged vibrissae among pinnipedimorphs in general, and the greatly enlarged foramen in *Odobenus* and other odobenids is probably associated with robust and tactile lips. Character 11 of Berta and Wyss [5], modified by Kohno ([1]: character 6).

7. Ventral tuberosity of zygomatic root. 0= absent. 1= present.

The anteroventral surface of the zygomatic 'root' of the maxilla is primitively smooth and concave (state 0) in terrestrial carnivores and Pinnipedimorpha (*Enaliarctos*). Many pinnipeds also exhibit the primitive condition (*Callorhinus*, desmatophocids, *Monachus*, *Imagotaria*, *Pontolis*, *Gomphotaria*). However, a small and convex or pointed tuberosity (state 1) is present in many early-diverging pinnipediforms and odobenids, including *Pteronarctos*, *Pacificotaria*, *Prototaria*, *Proneotherium*, as well as odobenines and *Dusignathus seftoni*. Character 22 of Kohno et al. [4], modified by Kohno ([1]: character 7).

8. Jugal: anteroventral process. 0= moderate. 1= long, reaching M1. 2= absent.

In pinnipedimorphs the jugal typically articulates to the maxilla along the anterior root of the zygomatic arch with an anteroventrally descending process, and a dorsal process along the ventral margin of the orbit; a zygomatic process of the maxilla divides these two prongs of the jugal. In the primitive condition that characterizes most pinnipedimorphs and all odobenids, the anteroventral process is of moderate length and does not approach the toothrow (state 0). Two derived conditions exist. In Desmatophocidae, the anteroventral process of the jugal is elongate and splint-like and nearly contacts the M^1^ (state 1). In phocids, the jugal lacks an anteroventral process (state 2). Character 8 of Kohno [1].

9. Palate. 0= flat. 1= arched transversely. 2= arched transversely and longitudinally.

Palatal morphology is somewhat variable within pinnipedimorphs, and is primitively flat (state 0) within the group, including "enaliarctines", *Callorhinus*, many phocids (e.g. *Erignathus* and *Monachus*), and early diverging odobenids (*Prototaria*, *Proneotherium*, *Neotherium*, and *Kamtschatarctos*. A transversely vaulted palate (state 1) characterizes later diverging “imagotariines” (*Imagotaria*, *Pseudotaria*, *Pontolis*) and *Aivukus*, while a palate that is both transversely and longitudinally arched is present in odobenines (*Protodobenus*, *Ontocetus*, *Valenictus*, *Odobenus*). A vaulted palate is associated with suction feeding in extant *Odobenus*, and the less derived transverse arching of some "imagotariines" and dusignathines may also be associated with suction feeding [2,6]. The palate of extant *Otaria* is also transversely arched, possibly indicating suction feeding behavior [2,6]. Character 9 of Deméré [2] and character 22 of Kohno [7], modified by Deméré and Berta ([8]: character 6) and Kohno ([1]: character 9).

10. Maxilla: palatal margins. 0= slightly divergent. 1= parallel. 2= posteriorly very wide.

A palate with slightly posteriorly diverging toothrows and lateral margins (state 0) is the primitive condition among carnivorans and pinnipedimorphs, and characterizes “enaliarctines” and odobenids in this study. Two derived conditions exist. *Callorhinus* and other Otariidae have parallel lateral margins of the palate (state 1). In Phocidae and Desmatophocidae, the toothrows diverge strongly posteriorly (state 2). Although we follow the coding of Kohno [1] for this character, we note that the palate of certain odobenids such as *Imagotaria* and *Pontolis* are nearly parallel and convergent with the otariid condition. In “enaliarctines” and terrestrial carnivores, posterior widening of the palate is perhaps related to the combined retention of a short snout and carnassials. Within desmatophocids (and *Allodesmus* in particular), this may be related to the extraordinarily large orbits and widely diverging temporal arches. Character 10 of Kohno [1], modified from character 13 of Berta and Wyss [5].

11. Palatine. 0= small. 1= long and posterolaterally expanded. 2= telescoped and underlying alisphenoid.

The palatine is primitively small (state 0) among pinnipedimorphs, including *Enaliarctos*, *Pteronarctos*, *Callorhinus*, Phocidae, *Prototaria*, and *Proneotherium*. Among most early diverging odobenids (including *Neotherium*, *Pseudotaria*, *Kamtschatarctos*, *Imagotaria*, and *Pontolis*) and dusignathines, the palatine is elongate in ventral aspect and posterolaterally expanded (state 1). In odobenines, the palatine has been ‘telescoped’ and ventrally obscures the alisphenoid (state 2). Character 11 of Kohno [1], modified from character 11 of Deméré [2].

12. Pterygoid: hamular process. 0= small and narrow. 1= large and broad.

In most pinnipedimorphs and terrestrial carnivores, the pterygoid hamulus is small, transversely narrow and delicate, and posteroventrally directed (state 0; or occasionally posterolaterally). In the Odobenini and *Protodobenus*, the hamular process is large, robust, and transversely wide (state 1). Character 12 of Deméré [2] and Kohno [1].

13. Pterygoid strut. 0= slender. 1= dorsoventrally thin and laterally projected. 2= dorsoventrally thick and laterally broad. 3= laterally thin and rolled.

The pterygoid strut is a horizontally oriented structure composed of the palatine, pterygoid, and alisphenoid lateral to the pterygoid hamulus and internal choanae along the posteromedial edge of the temporal fossa in ventral aspect. It is primitively slender (state 0) in *Enaliarctos*, *Pteronarctos*, *Callorhinus*, and *Pontolis*. The pterygoid strut is dorsoventrally thickened and transversely wide (state 1) in nearly all odobenids (excluding *Pontolis*). In odobenines, the strut is telescoped and obscured by the palatine in ventral view, presumably due to rostral foreshortening [2]. In desmatophocids, the strut is dorsoventrally thin but laterally directed (state 2 in [1]), and in phocids, the strut is transversely thin and rolled (state 3). Character 13 of Deméré [2], modified by Kohno ([1]: character 13).

14. Orbit: fossa muscularis. 0= present. 1= absent.

The fossa muscularis is a small depression on the dorsolateral surface of the maxilla, immediately anterior to the orbit. It is primitively present (state 0) in terrestrial carnivores and early diverging pinnipedimorphs like *Enaliarctos* and *Pteronarctos*. It is lost in all members of Pinnipedia (state 1). The inferior oblique muscle of the eye arises from this fossa. Character 8 of Berta and Wyss [5] and character 14 of Kohno [1].

15. Orbit: antorbital process. 0= small ridge on frontal. 1= prominent on frontal and maxilla. 2= prominent on maxilla only. 3= absent.

The antorbital process is a small flange-like process along the anteromedial margin of the orbit. It is primitively present as a small ridge on the frontal just posterior to the fossa muscularis and frontal-maxilla suture (state 0); this condition characterizes *Enaliarctos* and *Pteronarctos*. An antorbital process that is prominent and formed by both the maxilla and frontal (i.e. is bisected by the frontal-maxilla suture; state 1) is an odobenid synapomorphy and present in all odobenids for which this region is preserved. In otariids and phocids, the antorbital process is prominent but formed only on the maxilla (state 2); this condition was interpreted by Deméré [2] to be independently derived in these families. The antorbital process is not developed (state 3) in desmatophocids. Character 6 of Deméré [2]; modified by Kohno [1]. See also Berta and Wyss ([5]: character 16).

16. Frontal: supraorbital process. 0= small bump. 1= absent. 2= large and shelf like.

The supraorbital process is located dorsally on the frontal, overhanging the orbit. It is primitively present as a small bump in pinnipedimorphs (state 0) or as a small triangular projection in terrestrial caniforms. The primitive condition is present in early diverging odobenids (*Prototaria*, *Proneotherium*, *Pseudotaria*). It is absent in desmatophocids and phocids, *Neotherium*, *Imagotaria*, *Pontolis*, and dusignathine walruses. Enlarged, shelf-like supraorbital processes are a synapomorphy of otariids. Character 17 of Berta and Wyss [5]; modified by Kohno ([1]: character 16).

17. Frontal: interorbital bar. 0= moderate in width, broader than intertemporal region. 1= narrow, parallel sided. 2= very broad and short.

The morphology of the interorbital and intertemporal region varies in pinnipeds. In *Enaliarctos*, *Pteronarctos*, *Pinnarctidion*, *Prototaria*, and *Proneotherium*, the interorbital region is transversely wider than the intertemporal bar (state 0); this is equivalent to the condition in most terrestrial carnivores. Because of the presence of supraorbital shelves in otariids, *Callorhinus* also exhibits the primitive character state.

A narrow interorbital bar with parallel sides (state 1) is present in most pinniped groups, including Desmatophocidae, Phocidae, and many early diverging odobenids such as *Neotherium*, *Kamtschatarctos*, *Pseudotaria*, *Imagotaria*, and *Pontolis*. An anteroposteriorly short and broad interorbital bar (state 2) is present in the Dusignathinae + Odobeninae clade, and is related to the evolution of a shorter and broader cranium.

Character 17 of Kohno [1].

18. Jugal: postorbital process. 0= small. 1= dorsally projecting.

In most pinnipedimorphs, the postorbital process of the jugal is small, forming a subtriangular projection just anterior to the zygomatic process of the squamosal (state 0). This condition characterizes enaliarctines, Otariidae, early diverging odobenids, and dusignathines. A robust and dorsally projecting fingerlike postorbital process occurs in *Aivukus*, *Ontocetus*, *Valenictus*, and *Odobenus*. Character 18 of Kohno [1].

19. Zygomatic process. 0= long and slender. 1= short and slender. 2= dorsoventrally deep. 3= exaggerated.

The zygomatic process of the squamosal is primitively long and splint-like in pinnipedimorphs (state 0) such as *Enaliarctos*, *Pteronarctos*, and otariids. The more robust zygomatic process of odobenids is relatively short but still narrow (state 1), while in *Desmatophoca* and *Allodesmus*, the zygomatic process is dorsoventrally expanded (state 2). In extant phocids the zygomatic has an exaggerated dorsal expansion (state 3).

Character 6 of Kohno [7], modified by Deméré and Berta ([8]: character 9) and Kohno ([1]: character 19).

20. Orbital vacuity. 0= absent. 1= present/anteriorly positioned. 2= present/posteriorly positioned.

Unossified gaps in the medial wall of the orbit are variably developed in pinnipeds. The orbital wall is primitively solid among pinnipeds and terrestrial carnivores (state 0); pinnipedimorphs that exhibit the primitive character state include *Enaliarctos*, *Pteronarctos*, Desmatophocidae, *Erignathus*, and *Aivukus*. Two derived states are present. An unossified vacuity positioned anteriorly within the orbit (state 1) characterizes Otariidae, *Neotherium*, *Proneotherium*, *Prototaria*, *Pseudotaria*, *Dusignathus santacruzensis*, and *Gomphotaria*. A vacuity that is posteriorly positioned (state 2) is present in *Ontocetus*, *Valenictus*, and *Odobenus*. The development of an orbital vacuity is possibly associated with decreased bite force in pinnipeds as well as transverse thinning of the intertemporal region associated with increased orbit size. The posterior position of the vacuity in the Odobenini is possibly related to the development of wide rostrum housing large tusks, necessitating a stronger anterior orbital wall. Character 24 of Deméré [2] and character 20 of Kohno [1].

21. Orbit: optical foramen and orbitosphenoid. 0= plate-like. 1= funnel shaped.

The optical foramen is primitively situated within a platelike orbitosphenoid in the posterior part of the medial orbital wall; the orbitosphenoid extends anteriorly to the optical foramen (state 0). In the derived condition, the orbitosphenoid is funnel-shaped and does not extend far anterior to the foramen (state 1); this condition is present in the Odobenini (*Ontocetus*, *Valenictus*, *Odobenus*). The primitive condition is present in all other pinnipedimorphs in this analysis. Character 23 of Deméré [2], modified by Kohno ([1]: character 21).

22. Internal auditory meatus. 0= rounded. 1= bilobed. 2= canals for cranial nerves VII and VIII separated.

The internal auditory meatus serves as the cerebral entrance of the bony conduit for the facial and vestibulocochlear nerves (cranial nerves VII and VIII, respectively). In the primitive condition, represented in the current analysis by *Callorhinus*, the internal auditory meatus is round (state 0). A bilobate meatus (state 1) appears in all odobenids for which this feature is prepared, in addition to *Enaliarctos*, *Desmatophoca*, and *Allodesmus*. The second derived condition is present in Phocidae, in which the facial and vestibulocochlear nerve canals are separated (state 2). Although we follow the coding of Kohno [1], it should be noted that Berta and Wyss [5]also considered *Allodesmus* and *Pinnarctidion* to exhibit separated nerve canals (state 2). Character 22 of Kohno [1].

23. Epitympanic recess. 0= small. 1= large. 2= very large.

The epitympanic recess is the term given to the dorsal portion of the tympanic cavity. It is primitively small (state 0) in the early pinnipedimorphs *Enaliarctos* and *Pteronarctos*, as well as otariids (*Callorhinus*). An enlarged epitympanic recess (state 1) is present in all odobenids for which the structure is prepared. A greatly enlarged recess characterizes desmatophocids and phocids and may represent a phocoid synapomorphy. Character 23 of Kohno [1], modified from Kohno ([7]: character 2) and Kohno et al. ([4]: character 3).

24. Anterior opening of carotid canal. 0= proportionate. 1= retracted.

The carotid canal perforates the medial portion of the tympanic bulla in pinnipedimorphs, and the anterior opening of the carotid canal (=median lacerate foramen) is visible in ventral view at the anteromedial corner of the tympanic bulla in most pinnipedimorphs (state 0). In *Allodesmus* and *Desmatophoca*, the anterior opening of the canal is retracted and positioned within an embayment of the anteromedial corner of the tympanic bulla ([9]:123). The primitive condition characterizes all other pinnipedimorphs in our analysis. Character 24 of Kohno [1].

25. Squamosal fossa on zygomatic root. 0= present. 1= reduced.

A large fossa on the dorsal surface of the squamosal between the zygomatic process and the lateral wall of the braincase (state 0) is primitively present in pinnipedimorphs. In some otariids (*Callorhinus*) as well as odobenine walruses including *Aivukus*, *Ontocetus*, *Valenictus*, and *Odobenus*, the fossa is reduced, perhaps due to the development of a larger, more inflated braincase. Character 25 of Kohno [1]. Also see Deméré and Berta ([9]:144).

26. Glenoid fossa. 0= deep. 1= shallow. 2= laterally shortened.

The glenoid fossa on the squamosal is primitively deep (state 0) for a solid articulation with the mandibular condyle. The primitive condition is present in enaliarctines, *Callorhinus*, *Desmatophoca*, and several early diverging odobenids (*Prototaria*, *Proneotherium*, *Neotherium*, *Kamtschatarctos*, and *Pseudotaria*). A shallow glenoid fossa (state 1) characterizes *Allodesmus* and later diverging odobenids (*Imagotaria*, *Pontolis*, Dusignathinae, and Odobeninae). A second derived condition occurs in Phocidae, which is a transversely shortened glenoid fossa (state 2). Character 17 of Kohno et al. [4], character 18 of Kohno [7], and character 26 of Kohno [1].

27. Squamosal: transverse ridge. 0= absent. 1= present.

According to Kohno (1994), a transverse ridge is present in the squamosal fossa extending from the lateral side of the braincase to the base of the zygomatic process and the supramastoid crest (*sensu* Mead and Fordyce, 2009). Such a ridge is present only in *Prototaria planicephala* and *Prototaria primigena* [4,7]. Character 24 of Kohno et al. [4], character 11 of Deméré and Berta [8], and character 27 of Kohno [1].

28. Pseudosylvian sulcus. 0= present, deep. 1= reduced or absent.

In *Enaliarctos* and *Pteronarctos*, a well-defined vertical sulcus is present on the anterolateral surface of the braincase (state 0) corresponding to the pseudosylvian sulcus on the lateral side of the brain. This sulcus is present as a deep crease in the brain endocast of *Enaliarctos mealsi* [10], dividing the cerebral hemispheres roughly into anterior and posterior halves. The primitive state is also found in *Desmatophoca* and several early diverging odobenids (*Prototaria*, *Proneotherium*, *Neotherium*). All other pinnipeds in this study (including *Callorhinus* and phocids) lack an external expression of the pseudosylvian sulcus on the braincase (state 1). Modified from character 50 of Berta and Wyss [5] by Kohno [1].

29. Bony tentorium. 0= far from petrosal. 1= appressed to petrosal. 2= reduced.

The bony tentorium is a subhorizontal ridge on the inside of the braincase that is associated with the meninges and separates the cerebral hemispheres (dorsally) from the cerebellum (ventrally). It is primitively separated from the dorsal surface of the petrosal (state 0), a condition present in enaliarctines, *Callorhinus*, and Desmatophocidae. Two derived conditions are present. In modern *Odobenus* and all fossil odobenids where this region is prepared, the bony tentorium is closely appressed to the dorsal surface of the petrosal (state 1). In the second derived state, the bony tentorium is reduced (state 2); this characterizes phocids. Character 4 of Kohno [7], character 8 of Kohno et al. [4], and character 29 of Kohno [1].

30. Basioccipital. 0= narrow and parallel sided. 1= broad and pentagonal.

Among pinnipeds and odobenids, a narrow basioccipital with parallel lateral margins (state 0) is primitive, and is present in enaliarctines, *Callorhinus* and other otariids, Phocidae, *Desmatophoca*, and early diverging odobenids including *Prototaria*, *Proneotherium*, *Neotherium*, and *Pseudotaria*. A broad basioccipital with anteriorly converging lateral margins that give it a pentagonal outline in ventral aspect (state 1) is present in later diverging odobenids, such as *Imagotaria*, *Pontolis*, *Dusignathus*, *Gomphotaria*, and odobenines. Character 30 of Kohno [1], modified from character 14 of Deméré [2].

31. Posterior lacerate foramen. 0= round. 1= transversely expanded. 2= fissure.

The posterior lacerate foramen, present between the basioccipital, posterior squamosal, and tympanic bulla, is primitively round or circular in pinnipedimorphs (state 0); this condition is present in *Enaliarctos*, *Pteronarctos*, *Callorhinus*, *Desmatophoca*, and all odobenids where it is preserved. The foramen is transversely expanded (state 1) in *Allodesmus* and *Monachus* and expressed as a fissure in some phocids such as *Erignathus* (state 2). Berta and Wyss [5] considered an enlarged posterior lacerate foramen as a pinnipedimorphs synapomorphy, and that a posterior lacerate foramen which extended medially to the tympanic bulla was a synapomorphy of the Phocinae. Character 43 of Berta and Wyss [5], modified by Kohno ([1]: character 31).

32. Lambdoidal crest. 0= crestlike, posterodorsally directed. 1= flattened, anterodorsally directed.

The lambdoidal crest (=nuchal crest; Mead and Fordyce [11]) is an elongate crest formed along the parietal/occipital suture. It is primitively developed as a crest and dorsally or posterodorsally directed (state 0); in *Gomphotaria* and some otariids, it is formed as a high crest that can obscure the posterior braincase in dorsal aspect. The derived condition is present in *Pliopedia*, *Ontocetus*, *Valenictus*, and *Odobenus* [2], where the crest is anterodorsally directed and flattened; occasionally it is flattened into a crescent-shaped rugose facet (e.g. *Valenictus*). Character 18 of Deméré [2] and 32 of Kohno [1].

33. Mastoid process. 0= small. 1= large. 2= very large as widest part of skull. 3= pachyostotic.

The mastoid process is primitively small (state 0) among pinnipedimorphs, and appears as a small lateral projection of the squamosal just posterior to the glenoid fossa and external auditory meatus in *Enaliarctos*, *Pteronarctos*, *Callorhinus*, desmatophocids, and early diverging odobenids such as *Prototaria*, *Proneotherium*, *Neotherium*, *Pseudotaria*, and the dusignathine *Gomphotaria*. A larger mastoid process is present in *Imagotaria*, *Pontolis*, *Dusignathus*, and *Aivukus*. In the Odobenini, the mastoid process is massively inflated and extends laterally further than the lateral margin of the zygomatic arch, forming the widest part of the skull [2]. In phocids, the mastoid process is dense and pachyostotic (state 3). State 2 is paralleled in *Dusignathus seftoni*, where the widest part of the skull is formed by the mastoid process; this does not appear to characterize *Dusignathus santacruzensis* judging by the zygomatic and mastoid morphology of the holotype. *Dusignathus seftoni* was coded as state 1 (large mastoid) following Kohno [1] as the process is not grossly inflated as in *Ontocetus*, *Valenictus*, and *Odobenus*. Modified from characters 23 and 24 of Berta and Wyss [5], characters 15 and 16 of Deméré [2], by Kohno ([1]: character 33).

34. Paroccipital process. 0= small and separated from mastoid process. 1= enlarged posteriorly but still separate from mastoid process. 2= moderately sized and joined with mastoid process by a continuous ridge. 3= flattened and plate-like.

The morphology of the paroccipital process (=paramastoid process of Deméré, [2]) of the exoccipital is variable within pinnipedimorphs; the process is composed of the ventrolateral extremity of the exoccipital, and it is positioned immediately posterior to the mastoid process. The primitive condition is present in *Enaliarctos* and *Phocidae*, where the paroccipital process is small, delicate, and separated from the mastoid process (state 0). *Desmatophoca* and *Allodesmus* exhibit a greatly inflated and posterolaterally projecting paroccipital process that is still separated from the mastoid by a concave surface in ventral aspect (state 1). In *Pteronarctos*, *Callorhinus*, *Prototaria*, and *Proneotherium*, the paroccipital process projects posterolaterally, is relatively larger than in state 0 and similar in size to the mastoid process, and connected to it by a continuous ventrolateral ridge (state 2). In all later diverging odobenids, the paroccipital process is reduced to a flattened plate that is tightly appressed to the posterior surface of the mastoid process (state 3). Modified from character 17 of Deméré [2] and character 8 of Deméré and Berta [8] by Kohno ([1]: character 34).

35. Mandible: fusion of mandibular symphysis. 0= absent. 1= present.

Within the Pinnipedia, fusion of the mandibles at the symphysis only occurs amongst walruses. Five odobenids exhibit the derived condition (a fused symphysis): *Pelagiarctos thomasi*, *Dusignathus seftoni*, *Valenictus chulavistensis*, *Odobenus mandanoensis*, and *Odobenus rosmarus*. *Pelagiarctos* sp., *Dusignathus santacruzensis*, and all other fossil odobenids have unfused symphyses. Character 39 of Deméré [2].

36. Mandible: length of mandibular symphysis. 0=less than 50% of length of horizontal ramus. 1= greater than 50% of length of horizontal ramus.

This character describes the length of the mandibular symphysis relative to the length of the horizontal ramus, and the derived condition constitutes mandibles with mandibular symphyses that are greater than 50% of the length of the horizontal ramus. The derived condition characterizes *Dusignathus*, *Gomphotaria*, *Protodobenus*, *Ontocetus*, *Valenictus*, and *Odobenus*. *Pelagiarctos* could not be scored for this character. New character.

37. Mandible: genial tuberosity. 0= absent or indistinct. 1= present; developed as small tubercle or process on anterior portion of ramus. 2= present and well developed; extends well below ventral margin of ramus.

The presence and size of the genial tuberosity on the ventral margin of the horizontal ramus varies among pinnipeds. No apparent genial tuberosity is present in "enaliarctines" or *Proneotherium*, while a small tubercle is present in *Neotherium*, *Kamtschatarctos*, *Protodobenus*, *Valenictus*, and *Odobenus*. In some odobenids (*Pelagiarctos*, *Imagotaria*, *Pontolis*, *Dusignathus*, and *Ontocetus*) the genial tuberosity is well developed and extends far below the ventral margin of the horizontal ramus. In *Gomphotaria pugnax*, the genial tuberosity is poorly formed and present as rugose ridges posterolateral to the symphysis on each dentary, and was coded as 'absent' because it is not clearly defined in lateral view. New character.

38. Mandible: anterior portion of symphyseal region. 0= smooth, compact bone. 1=rugose, vascular bone.

In most pinnipeds (and carnivores in general), the cortical bone surface of the entire mandible is typically smooth. However, in certain large odobenids, the anterior end of the symphyseal region of the mandibles exhibits rugose and vascularized bone with many minute foramina. This condition is present in *Pelagiarctos*, *Pontolis*, *Gomphotaria*, *Dusignathus seftoni*, *Ontocetus*, *Valenictus chulavistensis*, and *Odobenus*. The derived condition is also present in adult males of large-bodied extant pinnipeds such as *Otaria byronia*, *Eumetopias jubatus*, and *Mirounga angustirostris*. Character 41 of Deméré [2].

39. Mandible: horizontal ramus. 0= horizontal. 1= upturned.

This character describes the orientation of the symphyseal portion of the mandible relative to the rest of the horizontal ramus. The derived condition characterizes *Valenictus* and *Ontocetus*, where the horizontal ramus is upturned and anterodorsally oriented relative to the ascending ramus. All other odobenids (including *Odobenus*, *Protodobenus*, and all early diverging odobenids) have a dorsal margin of the horizontal ramus that is horizontal. The primitive condition is present in *Pelagiarctos*. New character.

40. Mandible: mandibular furrow. 0= absent. 1= anterodorsal end of mandibles have a longitudinal furrow anteriorly.

This character describes the presence or absence of a longitudinal and transversely concave furrow running along the dorsal margin of the mandibular symphysis. The derived condition is exhibited by *Odobenus* and *Valenictus*. *Pelagiarctos* and all other odobenids have the primitive condition. New character.

41. Mandible: edentulous mandibular terminus. 0= absent. 1= present.

This character is defined as an edentulous anterior extremity of the symphyseal portion of the mandible. The anterior end of the mandible is anteriorly expanded and edentulous in *Ontocetus*, *Valenictus*, and *Odobenus*. In *Valenictus* and (occasionally) *Odobenus*, it is transversely expanded into a "spoon" shape. The presence of this character in *Ontocetus* suggests that it is decoupled from symphyseal fusion and incisor loss. *Pelagiarctos* exhibits the primitive condition. New character.

42. Mandible: posteroventral terminus of mandibular symphysis. 0= same level or anterior to P_1_ or P_2_. 1= posterior to the level of P_2_.

This character relates to the posterior extension of the mandibular symphysis, and whether it extends to the anterior premolars or posterior to the second lower premolar. The derived condition characterizes *Gomphotaria pugnax*, *Pontolis magnus*, *Dusignathus santacruzensis*, and *Ontocetus*. All other odobenids (notably including *Protodobenus*, *Dusignathus seftoni*, and *Odobenus*) exhibit the primitive condition. *Valenictus chulavistensis* was scored as a "?" because of the lack of dentition. *Pelagiarctos* exhibits the primitive condition. New character.

43. Mandible: depth of horizontal ramus. 0= deepest part of horizontal ramus posterior to mandibular symphysis. 1= deepest part of horizontal ramus at posteroventral terminus of symphysis.

This character relates to the position of the dorsoventrally deepest part of the horizontal ramus. In most Carnivora, the dorsoventrally deepest portion of the horizontal ramus is positioned posteriorly near the P_4_ and M_1_, and likely relates to the use of enlarged carnassial teeth for mastication. The primitive condition also characterizes *Enaliarctos* and *Pteronarctos*. However, in all modern and fossil pinnipeds (sensu Berta and Wyss [5]) the deepest part of the horizontal ramus is positioned at the mandibular symphysis, and the mandible becomes deeper anteriorly towards the symphysis. This probably relates to the loss of shearing carnassial teeth and the functional transition from a masticating dentition to a pierce-feeding dentition during early pinniped evolution [6]. *Pelagiarctos* (and all other odobenids) exhibit the derived condition. New character.

44. Mandible: mandibular arch. 0= nearly parallel. 1= sharply divergent.

Most pinnipeds are characterized by having mandibles that in dorsal view are nearly parallel or slightly divergent. However, some odobenids have widely divergent mandibles, likely resultant from the evolution of short and wide rostra. The derived condition (widely divergent mandibles) occurs in *Dusignathus*, *Protodobenus*, *Ontocetus*, *Valenictus* *chulavistensis*, and *Odobenus*. *Pelagiarctos* exhibits the primitive condition. Character 42 from Deméré [2].

45. Mandible: ventral border. 0= straight. 1= sinuous.

This character describes the shape of the ventral margin of the mandible. In most pinnipeds and odobenids, the ventral margin is straight (e.g. *Proneotherium*, *Neotherium*, *Pelagiarctos*, *Odobenus*). However, a sinuous ventral margin is present in *Dusignathus*, *Pontolis* *magnus*, and some specimens of *Imagotaria*. *Pelagiarctos* has a straight ventral border of the mandible. Character 43 from Deméré [2].

46. Mandible: enlarged digastric insertion. 0= absent. 1= present.

According to Berta and Wyss [5], this feature diagnoses only desmatophocids. This character has been modified for dusignathine and odobenine walruses. *Dusignathus* does not have a "bony flange" (=digastric insertion) that has a posterior projection as in desmatophocids, but the digastric insertion is elongate, and robust; well-developed digastric insertions also appear in *Pontolis*, *Odobenus*, *Valenictus*, and *Ontocetus*. A large digastric insertion also occurs in *Eumetopias*, *Otaria*, *Phocarctos*, and *Mirounga* (and some specimens of *Arctocephalus* *pusillus*). The digastric insertion is not enlarged in *Pelagiarctos* (state 0). Modified from character 52 of Berta and Wyss [5].

47. Mandible: mandibular condyle. 0 = at or slightly above level of tooth row. 1 = elevated above tooth row.

The position of the mandibular condyle is variable within pinnipeds, and within most pinnipeds and terrestrial carnivorans, the condyle is positioned at approximately the same level as the tooth row. However, in certain odobenids such as *Valenictus* and *Ontocetus*, the condyle is dorsally elevated above the tooth row. This character could not be coded for *Pelagiarctos*. The derived condition also characterizes Desmatophocidae [5]. Modified from Character 53 of Berta and Wyss [5].

48. Mandible: mandibular foramen. 0 = directed anteroventrally. 1 = directed anterodorsally.

This character refers to the direction of the anterior margin of the mandibular foramen on the medial surface of the mandible. In most odobenids, the mandibular foramen is directed anterodorsally, while in *Neotherium*, *Imagotaria*, and *Odobenus*, the mandibular foramen is directed anteroventrally. The derived condition was originally used by Berta and Deméré [12] to differentiate fossil *Callorhinus* from *Arctocephalus*. Character 9 of Berta and Deméré [12].

49. Mandible: medial shelf of angular process. 0= does not form medial shelf. 1= forms small medial shelf.

In most pinnipeds the angular process (= pterygoid process) is a small and tabular process situated along the posteroventral margin of the mandible. In derived odobenids, the angular process forms a small medial shelf. Such a shelf is absent in "enaliarctines", *Proneotherium*, *Neotherium*, *Imagotaria*, and is present in all other odobenids (where preserved). All modern otariids exhibit a medial shelf of the angular process, and *Callorhinus* *gilmorei* lacks this feature, unlike its extant congener [12]. Character 24 from Berta and Deméré [12] and character 51 of Berta and Wyss [5].

50. Mandible: base of coronoid process. 0= narrow (less than 42% of mandible length). 1= broad (greater than or equal to 42% of mandible length).

Most pinnipeds have coronoid processes that are relatively anteroposteriorly short. Two odobenids (*Pontolis* and *Odobenus*) have elongate coronoid processes that at least 42% of the length of the dentary. This character could not be coded for *Pelagiarctos*. New character.

51. Upper incisors: number. 0= three. 1= two. 2= one. 3= incisors absent.

The retention of three upper incisors per side is the primitive condition for pinnipeds and odobenids, and is present in Otariidae, *Enaliarctos*, *Pteronarctos*, most Phocidae (e.g. *Erignathus*), Desmatophocidae, and basal odobenids including *Prototaria*, *Proneotherium*, *Neotherium*, *Pontolis*, and *Protodobenus*. The loss of one incisor (typically the medial incisor) characterizes *Gomphotaria*, *Aivukus*, and *Ontocetus*. Some specimens of *Imagotaria* exhibit the primitive condition (three incisors) while one specimen (USNM 184060) bears only two incisors (derived state 1). A single incisor per side characterizes *Odobenus* (state 2), and *Valenictus* lacks incisors (state 3). Character 25 of Deméré [2].

52. Upper I^3^. 0= moderate. 1= long and slender. 2= premolariform. 3= absent.

A moderately proportioned upper third incisor is the primitive condition for pinnipeds and Odobenidae, while a long and slender incisor is present in *Gomphotaria* and Desmatophocidae (derived state 1). A reduced, premolariform incisor occurs in *Aivukus*, *Ontocetus*, and *Odobenus* (derived state 2) while *Valenictus* lacks upper incisors (state 3). Character 21 of Kohno [7]); modified by Kohno ([1]; character 42).

53. Lower incisors: number. 0= 2 incisors. 1= 1 incisor. 2= incisors absent.

The possession of three lower incisors per side is the primitive condition for arctoid carnivorans. *Enaliarctos* *emlongi* and *Pteronarctos* *goedertae* and most otariids and phocids have two lower incisors per side, and have lost the medial incisor. *Pontolis* and *Dusignathus* *seftoni* only have one lower incisor, while *Gomphotaria*, *Valenictus*, and *Odobenus* lack incisors altogether. The primitive character state is present in "enaliarctines", all other "imagotariines" *s. l.* (including *Pelagiarctos*), and *Dusignathus* *santacruzensis*. Character 60 from Berta and Wyss [5].

54. Lower incisors: relative size. 0= lower incisors of equal size, form transverse arcade. 1= lateral incisors greater in size than medial incisors, medial incisor placed posteromedial to lateral incisor.

Arctoid carnivorans (and pinnipeds) primitively exhibit lower incisors that are of equal size and are arrayed in an arcade. In more derived forms, such as some odobenids and "otariine" sea lions, the lateral incisor is enlarged while the small medial incisor is positioned posteromedially; this makes the incisor alveoli appear 'V' shaped. In *Eumetopias* *jubatus* the medial incisor is more anteriorly oriented than the lateral incisor to compensate for its posterior position, and the incisor crowns still form an arcade. *Neotherium* and "enaliarctines" are characterized by the primitive condition (state 0). *Pelagiarctos* exhibits lateral incisors with roots that are only slightly wider than medial incisors, but the medial incisors are posteriorly positioned (state 1). *Imagotaria*, *Dusignathus* *santacruzensis*, *Ontocetus*, and *Protodobenus* exhibit the derived condition (state 1); *Proneotherium*, *Kamtschatarctos*, *Dusignathus* *seftoni*, *Gomphotaria*, *Valenictus*, and *Odobenus* could not be coded for this character because of the lack of preserved incisors or well preserved alveoli, loss of medial incisors, or loss of both lower incisors. Modified from character 132 of Bininda-Emonds and Russell [13].

55. C^1^. 0= proportional. 1= tusk like. 2= tusk like with globular osteodentine.

In most pinnipeds and odobenids, the upper canine is proportionately sized and lacks globular dentine (primitive state). Tusk-like upper canines that lack globular dentine are present in *Gomphotaria* and *Dusignathus*. Globular dentine is a dental tissue that is unique to odobenine walruses with presumed ever-growing tusks [2]) and is present in *Odobenus*, *Valenictus*, *Ontocetus*, and *Protodobenus*. Modified from characters 27-28 of Deméré [2] by Kohno ([1]).

56. C_1_: size compared to C^1^. 0= nearly equal (100-80%) and caniniform. 1= reduced (75-20%) and premolariform. 2= lower canine absent.

Upper and lower canines are primitively similar in size amongst arctoid carnivorans and pinnipeds. Odobenids primitively have caniniform canines. Odobenine walruses, however, are characterized by having low-crowned, premolariform lower canines that are reduced in size, evolving with enlarged upper canines [2]. Reduction of lower canine size occurs within *Protodobenus*, *Ontocetus*, and *Odobenus*, which possess state 1 (a lower canine that is less than 75% of the width of the upper canine). Further reduction occurs in *Valenictus*, which has lost the lower canine (state 2). Although upper canines are unknown for *Pelagiarctos*, its lower canine is caniniform and thus represents the primitive condition. Modified from characters 28 and 31 of Deméré [2].

57. C_1_: posterior crista. 0= present. 1= absent.

A posterior crista (=crest or carina) is primitively present on arctoid carnivoran and pinniped canines. Barnes and Raschke [14] noted that a posterior crista was lacking on the lower canines of *Dusignathus* *santacruzensis* and *Gomphotaria*, uniting them within the Dusignathinae. A new specimen of *Dusignathus* *seftoni* also lacks a posterior crista, and so does *Pontolis* *magnus*; in these taxa, this is related to the enlargement and tusk-like development of the lower canines. However, we note that a posterior crista is also absent in odobenines such as *Protodobenus*, *Ontocetus*, and *Odobenus*. *Pelagiarctos*, "enaliarctines", *Proneotherium*, *Neotherium*, and *Imagotaria* all exhibit the primitive condition. New character.

58. C_1_: longitudinal fluting. 0= absent. 1= present.

Most carnivorans and pinnipeds exhibit smooth enamel on their canines. However, the lower canines of *Gomphotaria* and *Dusignathus* *seftoni* have elongate, longitudinal grooves or 'fluting' in the surface of the tooth. Longitudinal fluting also characterizes upper canines of *Ontocetus* and some specimens of *Valenictus* and *Odobenus*. *Pelagiarctos* has smooth enamel on its lower canines. In *Gomphotaria*, the postcanines and upper incisors are also longitudinally fluted. New character.

59. C_1_: root. 0= oval or circular in cross section. 1= bilobate in cross section or prominent longitudinal labial sulcus.

Most pinnipeds and other carnivorans have lower canines with a circular or oval-shaped cross section. The canines of *Pelagiarctos* and *Imagotaria* have longitudinal sulci that give the tooth a bilobate cross section [15]. The derived condition is present in the holotype of *Imagotaria* *downsi*, but is less evident in referred material from the Santa Margarita Sandstone (USNM 23858) and UCMP 88459) because the canines are only partially erupted. New character.

60. C_1_: orientation. 0= not procumbent and vertically oriented. 1= procumbent.

Pinnipeds and arctoid carnivorans primitively have canines that are vertically oriented. *Dusignathus* *santacruzensis*, *Dusignathus* *seftoni*, and *Gomphotaria*, on the other hand, have lower canines that are procumbent and are directed anterodorsally. New character.

61. Lower premolars. 0= P_1-4_ present. 1= P_1-3_ present, P_4_ absent. 2= premolars absent.

Arctoid carnivorans and pinnipeds primitively possess all four lower premolars. Dental reduction in odobenins has resulted in the loss of the P_4_ in *Odobenus* and loss of all premolars in *Valenictus*. New character.

62. Postcanines: tooth enamel. 0= well developed. 1= thin or patchy. 2= enamel absent in postcanine teeth of adults.

Well developed enamel on postcanine teeth is primitive for carnivorans and pinnipeds. Within odobenids, enamel loss and reduction is observed: thin and patchy enamel is present in *Dusignathus* *santacruzensis*, *Aivukus*, *Protodobenus*, *Pontolis*, and *Gomphotaria*, while enamel is absent in adult postcanine teeth of *Ontocetus* and *Odobenus*. Due to the complete loss of postcanines, *Valenictus* was scored as a '?' for this character. *Pelagiarctos* retains thick postcanine tooth enamel (state 0). Modified from character 38 of Deméré [2].

63. Postcanines: crowns. 0= laterally compressed. 1= bulbous.

Pinnipeds primitively exhibit postcanine tooth crowns that are linguo-labially compressed and often triangular in labial aspect. The derived condition - inflated and bulbous crowns - occurs in *Pelagiarctos*, *Imagotaria*, *Dusignathus*, *Pontolis*, *Protodobenus*, *Ontocetus*, and *Odobenus*. Berta and Wyss [5] note that it occurs in *Allodesmus*, *Desmatophoca*, and some phocids (e.g. *Mirounga*, *Halichoerus*). Character 75 of Berta and Wyss [5].

64. Postcanines: root lobes. 0= root lobes of postcanine teeth of equal or narrower width than crowns. 1= root lobes of postcanine teeth inflated and wider than crowns.

Most pinnipeds and arctoid carnivorans have postcanine tooth roots that are relatively narrow and finger-like. However, *Pontolis*, *Dusignathus* *santacruzensis*, and *Gomphotaria* have postcanine teeth with roots that are inflated and in labial or lingual aspect, wider than their tooth crowns. Barnes and Raschke [14] considered this addition of cementum a dusignathine character. New character.

65. Lower postcanines: tooth row length. 0= long (length of tooth row greater than 40% of the mandible length). 1= short (length of tooth row less than or equal to 40% of the mandible length).

Pinnipediforms are characterized by having a short postcanine toothrow, which is related to the anterior crowding of teeth in the mandible [6,16]. Berta and Wyss [5] originally considered only *Pteronarctos*, *Callorhinus*, and *Otaria* to exhibit the derived condition, but Adam and Berta [6] noted that the M_1_ is positioned anteriorly to the halfway point of the mandible in *Pteronarctos* and all later diverging pinnipediforms (but not *Enaliarctos*). We measured postcanine tooth length and mandible length. *Enaliarctos* has a tooth row 49% of the mandible length, while all other pinnipedimorph taxa have a toothrow < 40% of the mandible length (~25% in *Ontocetus* as the shortest, and 36% in *Proneotherium* as the longest). *Enaliarctos* is characterized by the primitive condition, and all other taxa in this analysis are characterized by the derived condition. Because *Pelagiarctos* does not have a complete mandible it could not be scored. Modified from character 69 of Berta and Wyss [5].

66. Lower postcanines: paraconid cusps. 0= present and well developed. 1= absent or reduced.

Paraconid cusps (=anterior cusps of Berta and Wyss, [5]) are present in most pinnipeds, and in many "imagotariine" walruses. Berta and Wyss [5] initially considered this character to only be present in *Enaliarctos* and *Desmatophoca*, but not terrestrial arctoids, or other pinnipeds. We modified this character to represent a wider range of taxa which have well developed (but not necessarily large) paraconid cusps (state 0). Many stem odobenids exhibit minor (but distinct) paraconids (also state 0), while odobenines and dusignathines appear to lack this feature (state 1). The primitive condition is present in *Pelagiarctos*. Modified from character 70 of Berta and Wyss [5].

67. Lower postcanines: lingual cingula. 0= present and smooth. 1= present and rough or crenulated.

A smooth lingual cingulum is present in most odobenids, phocids, and some otariids (*Callorhinus* and some *Arctophoca*). In this study, a smooth lingual cingulum characterize *Enaliarctos*, *Pteronarctos*, *Proneotherium*, *Neotherium*, *Pontolis*, *Protodobenus*, and *Dusignathus*. A cingulum that is crenulated with small cusps is present in *Imagotaria* and *Pelagiarctos* (state 1). Crenulated lingual cingulum also occurs in some otariids. New character.

68. Lower premolars: hypoconid cusps. 0= present. 1= absent.

Hypoconid cusps (occasionally referred to as posterior accessory cusps) are primitively present in most pinnipeds. Hypoconid cusps are present in "enaliarctines" and most "Imagotariinae" *s. l*., including *Pontolis* *magnus*. *Imagotaria* *downsi* exhibits variable presence of a hypoconid, and they are typically present on the M_1_ but not the P_1-4_ (e.g. USNM 23858). For this reason, *Imagotaria* was coded as possessing both states. Large hypoconids are present in *Pelagiarctos*. Hypoconids are lost in *Dusignathus*, *Protodobenus*, *Ontocetus*, and *Odobenus*. New character.

69. Lower postcanines: talonid basin. 0= absent. 1= slight concavity or small shelf.

The talonid basin (= posterolingual shelf of Deméré and Berta [8]) is absent in "enaliarctines" [17] and early "imagotariines" *s.l*. However, we noted that some odobenids including *Pelagiarctos*, *Imagotaria*, *Kamtschatarctos*, *Pontolis*, and *Dusignathus* *santacruzensis* exhibit a slight concavity or shelf (derived condition, state 1) on the posterolingual margin of lower postcanine teeth, which we tentatively interpret as redevelopment of a talonid basin. New Character.

70. Lower postcanines: metaconid. 0= present. 1= reduced. 2= absent.

The metaconid cusp is located on the posterior crista between the protoconid (principal cusp) and hypoconid cusp (posterior cusp), and is primitively present in pinnipedimorphs. Berta and Wyss [5] considered the metaconid cusp to be present in terrestrial arctoids, reduced in *Enaliarctos* and *Pteronarctos*, and absent in all later diverging pinnipeds. However, we noted that well developed metaconid cusps are present in *Enaliarctos*, *Pteronarctos*, and *Proneotherium*. We modified this character to reflect the variation of metaconid morphology within Pinnipedia and the Odobenidae. The metaconid is reduced (relative to *Proneotherium* and "enaliarctines") in *Pelagiarctos*, *Neotherium*, and *Kamtschatarctos*. The metaconid is lost in *Imagotaria*, *Pontolis*, *Dusignathus* *santacruzensis*, *Gomphotaria*, *Protodobenus*, *Ontocetus*, and *Odobenus*. Metaconid cusps are completely absent in all described fossil and modern otariids. Well developed metaconid cusps are present in *Desmatophoca* and many phocid seals (e.g. *Phoca*, *Pusa*, *Lobodon*), and absent in some (e.g. *Mirounga*). Modified from character 71 of Berta and Wyss [5].

71. P^1-2^, lingual cingulum. 0= distinct but small. 1= well developed with cuspules. 2= weak and bulbous. 3= P^1-2^ absent.

The lingual cingulum (a low enamel ridge along the linguo-basal margin of the crown) is variably developed among pinnipeds. *Enaliarctos*, *Pteronarctos*, Otariidae, and Phocidae typically exhibit the primitive condition, while well-developed cingula with cuspules (derived state 1; or crenations; [18]) are present in early diverging odobenids (*Prototaria*, *Proneotherium*, *Neotherium*, *Imagotaria*, and *Pontolis*). Weakly developed cingula present as a mild inflation or ridge on bulbous cheek teeth characterize *Dusignathus*, *Gomphotaria*, and all odobenines except *Valenictus*, which lacks postcanine teeth (state 3). The derived character states are also found in *Desmatophoca* (strong cingula with cuspules) and *Allodesmus* (weakly developed, bulbous cingula). Character 45 of Kohno [1].

72. P_2_: rooting. 0= double or bilobate. 1= single.

Although the postcanine teeth of arctoid carnivorans are primitively double-rooted (with the exception of the P_1_), a transition towards single-rootedness has occurred independently several times within pinnipeds, and in each case typically beginning from the anterior and progressing to the posterior postcanines [16]. Root fusion began with the P_2_ in otariids [12,16,19](Repenning and Tedford, 1977; Berta and Deméré, 1986; Boessenecker, 2011), and appears to have evolved in parallel in Odobenidae [2,19,20] and Desmatophocidae [20]. Most "Imagotariinae" *s. l*. have a double-rooted P_2_. Like referred specimens of *Pelagiarctos* *thomasi*, *Pelagiarctos* sp. also exhibits a bilobate P_2_; because few other odobenid taxa possess bilobate P_2_, we included bilobate roots in the primitive character state. A single-rooted P_2_ (state 1) is present in *Dusignathus* *santacruzensis*, *Dusignathus* *seftoni*, *Gomphotaria*, *Protodobenus*, *Ontocetus*, and *Odobenus*. *Imagotaria downsi* preserves individuals with both double- and single-rooted teeth; a similar pattern of dental variation is documented in the Pliocene otariid *Callorhinus gilmorei* [12]. Modified from character 6 of Berta and Deméré [12].

73. P_3-4_: rooting. 0= double. 1= single.

As discussed in character 72 above, root fusion begins with the P_2_ and progresses posteriorly. A double-rooted P_3-4_ is present in "enaliarctines", *Proneotherium*, *Neotherium*, *Pelagiarctos*, *Kamtschatarctos*, and *Pontolis*. A single-rooted P_3-4_ is present *Dusignathus*, *Gomphotaria*, *Protodobenus*, *Aivukus*, *Ontocetus*, and *Odobenus*; it is present in some specimens of *Imagotaria* *downsi*, which was coded as possessing both states. Character 7 of Berta and Deméré [12].

74. P^3^: roots. 0= double rooted. 1= single and bilobed. 2= single and cylindrical. 3= P3 absent.

Similar to the lower dentition, the roots of upper postcanine teeth are primitively double or triple rooted, and single rooted in derived pinnipeds. Root fusion generally follows a similar pattern of cusp simplification in odobenids and other pinnipedimorphs.

The upper P^3^ is primitively double-rooted in *Enaliarctos*, *Pteronarctos*, most Phocidae (except *Mirounga*), some fossil Otariidae (*Pithanotaria*, *Thalassoleon*), and basal odobenids including *Prototaria*, *Proneotherium*, *Neotherium*, and *Pseudotaria*. A single but bilobate root (derived state 1) is present in *Imagotaria* and *Pontolis*; this reflects an intermediate grade of dental evolution between unfused and fully fused roots. Fully fused roots with a cylindrical shape (derived state 2) are present in *Dusignathus*, *Gomphotaria*, and all odobenines except *Valenictus*, which lacks upper postcanines (derived state 3). Only one possible reversal of this character has been noted among fossil pinnipeds: the “enaliarctine” *Pacificotaria hadromma* has a nearly triple-rooted P^2-3^, and Barnes (1992) hypothesized that the posterior roots had widened and become bilobate due to the apomorphic appearance of protocone cusps on the P^2-3^. Character 62 of Berta and Wyss [5] and character 46 of Kohno [1].

75. P^4^, protocone shelf. 0= anteromedially placed. 1= posteromedially placed with small cuspules. 2= reduced or absent.

A well developed and anteromedially positioned protocone shelf (state 0) is primitively present in *Enaliarctos*, and is associated with the retention of a carnassial; this feature unites early pinnipedimorphs with terrestrial carnivorans. A prominent but posteromedially placed protocone shelf with cuspules (state 1) characterizes *Pteronarctos*, *Pacificotaria*, *Pinnarctidion*, *Proneotherium*, *Imagotaria*, and *Neotherium*. The protocone shelf is reduced or completely absent (state 2) in Otariidae, Phocidae, Desmatophocidae, *Dusignathus*, *Gomphotaria*, and odobenines. Modified by Kohno ([1]: character 47) from Deméré ([2]: character 36) and Deméré and Berta ([8]: character 17).

76. P^4^, roots. 0= triple rooted. 1= double rooted. 2= single rooted. 3= P^4^ absent.

Being the upper carnassial, the P^4^ is primitively triple-rooted (state 0) in carnivores and pinnipedimorphs, including *Enaliarctos* and *Pinnarctidion bishopi*; a single anterior root is present, and two posterior roots are present. Root fusion in pinnipedimorphs began with the fusion of the two posterior roots of the P^4^ [21], resulting in a double rooted condition (state 1) in *Pteronarctos*, Phocidae, *Desmatophoca*, fossil Otariidae, and early diverging odobenids including *Prototaria*, *Proneotherium*, *Neotherium*, *Pseudotaria*, and *Imagotaria*. A single rooted P^4^ (state 2) is present in *Dusignathus* *seftoni*, *Gomphotaria*, and all odobenines except *Valenictus* which lacks a P^4^ (state 3). As mentioned above, root fusion is generally correlated with simplifying crown morphology, and is possibly a mode of increasing functional gape in pinnipeds [16]. Character 48 of Kohno [1], character 37 of Deméré [2] and character 64 of Berta and Wyss [5].

77. M^1^: roots. 0= triple rooted. 1= double rooted. 2= single rooted. 3= M1 absent.

The primitive condition characterizes the large molars of terrestrial carnivores, and is present in early diverging odobenids including *Prototaria*, *Proneotherium*, *Neotherium*, and *Kamtschatarctos*. Curiously, “enaliarctines” such as *Enaliarctos*, *Pteronarctos*, *Pacificotaria*, and *Pinnarctidion* exhibit a double rooted M^1^ (state 1), and are more dentally derived than early "imagotariines" with respect to this character. A double-rooted M^1^ also appears in *Pseudotaria*, *Imagotaria*, *Pontolis*, fossil Otariidae, and most Phocidae. A single-rooted M^1^ (state 2) occurs in extant Otariidae, *Mirounga*, *Dusignathus seftoni*, *Gomphotaria*, *Aivukus*, *Protodobenus*, and *Ontocetus*. The M^1^ of *Desmatophoca oregonensis* is most frequently double-rooted, but a single specimen exhibits a single-rooted M^1^ [9]. The M^1^ is absent in *Odobenus* and *Valenictus* (state 3). In a sample of modern *Callorhinus* *ursinus*, the M^1^ exhibited the greatest variation in root condition and was of all teeth the most frequently unfused (Boessenecker, 2011); this perhaps reflects the triple-rooted early history of this tooth in pinnipedimorphs. Character 65 of Berta and Wyss [5] and character 49 of Kohno [1].

78. M_1_. 0= present. 1= absent.

The M_1_ is present in nearly all arctoid carnivores and pinnipeds. Loss of the M_1_ occurs within the Odobeninae, and is absent in *Protodobenus*, *Ontocetus*, *Odobenus*, and *Valenictus*. The m1 is present in "enaliarctines", all "imagotariines" *s. l*., *Aivukus* (basal Odobeninae), and all otariids and phocids. New character.

79. M_1_: rooting. 0= double rooted. 1= single rooted.

As described above in characters 35 and 36, postcanine root fusion initiated with the P_2_ and progressed posteriorly, with the M_1_ being the last postcanine to exhibit fuse roots. A double-rooted M_1_ is present in "enaliarctines" and all "imagotariines" *s. l*., while a single-rooted M_1_ is present in *Dusignathus* spp., *Gomphotaria*, and *Aivukus*. *Protodobenus*, *Ontocetus*, *Valenictus*, and *Odobenus* could not be scored for this character due to the lack of an M_1_. Most phocid seals retain a double-rooted M_1_ (except for *Mirounga*). New character.

80. M^2^: rooting. 0= double rooted. 1= single rooted. 2= M^2^ absent.

This character shows an unusual distribution, similar to character 77 (M^1^:rooting). The M^2^ is primitively double-rooted (state 0) in early diverging odobenids such as *Neotherium*, *Pseudotaria*, *Kamtschatarctos*, and *Pontolis*. However, in the most primitive odobenids such as *Prototaria* and *Proneotherium*, as well as enaliarctines, the M^2^ is single rooted. The M^2^ is similarly single rooted in extant Otariidae, Desmatophocidae, *Dusignathus* *seftoni*, and *Gomphotaria*; *Imagotaria* is polymorphic and includes specimens with double and single rooted M^2^. An M^2^ is missing in Odobeninae and Phocidae. It is possible that development of a double rooted M^2^ in odobenids is analogous to root development in *Pacificotaria* [22], and may be an apomorphic feature that first appeared in the *Neotherium*-grade of walrus evolution. Character 13 of Kohno (1994) and character 50 of Kohno [1].

81. M_2_. 0= present. 1= absent.

The lower second molar is primitively present and single-rooted in geologically early pinnipeds and "enaliarctines". Among odobenids, an M_2_ is retained in *Proneotherium*, *Neotherium*, *Pontolis*, *Kamtschatarctos*, *Pelagiarctos*, and some specimens of *Imagotaria* *downsi*, while it is lost in all dusignathines and odobenines. The M_2_ is present in *Desmatophoca* and *Allodesmus* [9] but is lost in all known fossil and modern otariids (except possibly *Pithanotaria* *starri*; [19]). Modified from character 73 of Berta and Wyss [5].

82. Postcanine tooth wear. 0= absent. 1= minor wear on anterior and posterior cusps or apical wear. 2= large wear facets on anterior and posterior edges. 3= heavily worn and polished.

Tooth wear is variable among pinnipeds and arctoid carnivores. Tooth wear is generally light or absent (state 0) in "enaliarctines", whereas apical wear (state 1) occurs in most "imagotariines" (including *Pelagiarctos*), *Protodobenus*, and *Ontocetus*. Two other derived states exist: the presence of large wear facets on the anterior and posterior sides of the teeth (state 2), which characterizes *Pontolis* and *Dusignathus* *santacruzensis*, and teeth that are worn nearly flat (state 3), which characterizes *Odobenus* and *Gomphotaria*. New character.

83. Humerus: deltoid tubercle. 0= on pectoral crest. 1= on lateral edge of crest. 2= off crest.

The deltoid insertion tubercle is primitively positioned on the deltopectoral crest in pinnipeds and odobenids. The primitive condition occurs in *Neotherium*, *Imagotaria*, *Pontolis*, and *Aivukus*, while derived state 1 characterizes *Ontocetus*. Derived state 2 is present in *Odobenus*, *Valenictus*, and *Pliopedia*. Character 53 of Kohno [1] based on character 45 of Deméré [2].

84. Humerus: diameter of distal trochlea. 0= medial lip same diameter (or smaller) as distal capitulum. 1= medial lip diameter greater than distal capitulum.

The medial lip of the distal trochlea of the humerus is primitively similar or smaller in anteroposterior diameter to the distal capitulum in?. However, the derived condition characterizes all modern and fossil odobenids for which humeri are known [19]. Character 54 of Kohno [1] based on character 47 of Deméré [2].

85. Radius: distal end. 0= unexpanded. 1= expanded, with small radial process. 2= expanded, with large radial process.

The radii of carnivorans is primitively unexpanded; the primitive condition is present in *Enaliarctos*. Two derived conditions are present; the first – expanded distal radius with a small radial process – characterizes Otariidae and *Allodesmus*. An expanded distal end with a large and distally directed radial process characterizes all modern and fossil odobenids for which radii are known. Character 55 of Kohno [1] based on character 28 of Deméré [2].

86. Metacarpal I: insertion of pollicle extensor. 0= smooth. 1= pit. 2= rugosity.

The pollicle extensor muscle inserts on the dorsal proximal surface of the Metacarpal I, and is primitively smooth among pinnipeds. A pit (derived state 1) is present in *Neotherium*, *Imagotaria*, *Pliopedia*, and *Gomphotaria* while a distinct rugosity (derived state 2) is present in *Aivukus*, *Valenictus*, *Odobenus*, and *Ontocetus*. Character 56 of Kohno [1] based on character 49 of Deméré [2].

87. Scapholunar. 0= no pit for magnum. 1= well-formed pit.

The scapholunar primitively lacks a pit for the articulation with the magnum among pinnipeds. In *Odobenus* and fossil walruses for which a scapholunar is known, a well defined pit is present on the distal surface for the magnum articulation [19]. Character 57 of Kohno [1] based on character 50 of Deméré [2].

88. Astragalus: calcaneal process. 0= absent. 1= present. 2= elongated.

A calcaneal process on the posteromedial margin of the astragalus is primitively not developed in pinnipeds. Two derived conditions exist: a moderately developed calcaneal process is present in *Allodesmus* and Odobenidae (derived state 1), while an elongate and posteriorly directed process is present in Phocidae (derived state 2). Character 58 of Kohno [1] based on character 51 of Deméré [2].

89. Calcaneum: calcaneal tuber. 0= straight. 1= medially prominent.

Within pinnipeds, a calcaneum with parallel medial and lateral sides of the calcaneal tuber is primitive, and characterizes *Enaliarctos mealsi*, Otariidae, and Phocidae. The derived condition – a medially prominent calcaneal tuber – is present in *Allodesmus*, *Odobenus*, and fossil odobenids for which calcanea are known (*Proneotherium*, *Neotherium*, *Imagotaria*, *Pontolis*, *Dusignathus*, *Gomphotaria*, *Ontocetus*, *Valenictus*). Character 59 of Kohno [1] based on character 52 of Deméré [2].

90. Entocuneiform/mesocuneiform articulation. 0= abutting. 1= overlapping.

Primitively among pinnipeds, the articulation of the entocuneiform and mesocuneiform is straight, and at a 90˚ angle to the navicular articular facet. In *Allodesmus*, *Proneotherium*, *Pontolis*, *Gomphotaria*, and *Odobenus*, the two bones overlap. Additionally, the articular surface on the entocuneiform is nearly subparallel with the navicular articular surface, and the distal articular surface of the entocuneiform is large and convex. Character 60 of Kohno [1] based on character 53 of Deméré [2].

**References**

1. Kohno N (2006) A new Miocene odobenid (Mammalia: Carnivora) from Hokkaido, Japan, and its implications for odobenid phylogeny. Journal of Vertebrate Paleontology 26: 411-421.

2. Deméré TA (1994) The family Odobenidae: a phylogenetic analysis of living and fossil forms. In: Berta A, Deméré TA, editors. Contributions in Marine Mammal Paleontology honoring Frank C Whitmore, Jr: Proceedings of the San Diego Society of Natural History. pp. 99-123.

3. Mitchell ED (1966) The Miocene pinniped *Allodesmus*. University of California Publications in Geological Sciences 61: 1-46.

4. Kohno N, Barnes LG, Hirota K (1995) Miocene fossil pinnipeds of the genera *Prototaria* and *Neotherium* (Carnivora; Otariidae; Imagotariinae) in the North Pacific Ocean: evolution, relationships, and distribution. The Island Arc 3: 285-308.

5. Berta A, Wyss AR (1994) Pinniped Phylogeny. In: Berta A, Deméré TA, editors. Contributions in Marine Mammal Paleontology Honoring Frank C Whitmore, Jr: Proceedings of the San Diego Society of Natural History. pp. 33-56.

6. Adam PJ, Berta A (2002) Evolution of prey capture strategies and diet in the Pinnipedimorpha (Mammalia, Carnivora). Oryctos 4: 83-107.

7. Kohno N (1994) A new Miocene pinniped in the genus *Prototaria* (Carnivora: Odobenidae) from the Moniwa Formation, Miyagi, Japan. Journal of Vertebrate Paleontology 13: 414-426.

8. Deméré TA, Berta A (2001) A reevaluation of *Proneotherium repenningi* from the Miocene Astoria Formation of Oregon and it's position as a basal odobenid (Pinnipedia: Mammalia). Journal of Vertebrate Paleontology 21: 279-310.

9. Deméré TA, Berta A (2002) The Miocene pinniped *Desmatophoca oregonensis* Condon 1906 (Mammalia: Carnivora) from the Astoria Formation, Oregon. Smithsonian Contributions to Paleobiology 93: 113-148.

10. Mitchell ED, Tedford RH (1973) The Enaliarctinae: a new group of extinct aquatic Carnivora and a consideration of the origin of the Otariidae. Bulletin of the American Museum of Natural History 151: 203-284.

11. Mead JG, Fordyce RE (2009) The therian skull: a lexicon with emphasis on the odontocetes. Smithsonian Contributions to Biology 627: 1-248.

12. Berta A, Deméré TA (1986) *Callorhinus gilmorei* n. sp., (Carnivora: Otariidae) from the San Diego Formation (Blancan) and its implications for otariid phylogeny. Transactions of the San Diego Society of Natural History 21: 111-126.

13. Bininda-Emonds ORP, Russell AP (1996) A morphological perspective on the phylogenetic relationships of the extant phocid seals (Mammalia: Carnivora: Phocidae). Bonner, Zoologische Monographien 41: 1-256.

14. Barnes LG, Raschke RE (1991) *Gomphotaria pugnax*, a new genus and species of Late Miocene dusignathine otariid pinniped (Mammalia: Carnivora) from California. Contributions in Science, Natural History Museum of Los Angeles County 426: 1-16.

15. Barnes LG (1988) A new fossil pinniped (Mammalia: Otariidae) from the middle Miocene Sharktooth Hill Bonebed, California. Contributions in Science, Natural History Museum of Los Angeles County 396: 1-11.

16. Boessenecker RW (2011) New records of the fur seal *Callorhinus* (Carnivora: Otariidae) from the Plio-Pleistocene Rio Dell Formation of Northern California and comments on otariid dental evolution. Journal of Vertebrate Paleontology 31: 454-467.

17. Berta A (1991) New *Enaliarctos** (Pinnipedimorpha) from the Oligocene and Miocene of Oregon and the role of "Enaliarctids" in pinniped phylogeny. Smithsonian Contributions to Paleobiology 69: 1-33.

18. Deméré TA, Berta A (2005) New skeletal material of *Thalassoleon* (Otariidae: Pinnipedia) from the Late Miocene-Early Pliocene (Hemphillian) of California. Bulletin of the Florida Museum of Natural History 45: 379-411.

19. Repenning CA, Tedford RH (1977) Otarioid seals of the Neogene. Geological Survey Professional Paper 992: 1-87.

20. Mitchell ED (1975) Parallelism and convergence in the evolution of Otariidae and Phocidae. Rapports et procès-verbaux des réunions / Conseil permanent international pour l'exploration de la mer 169: 12-26.

21. Barnes LG (1989) A new enaliarctine pinniped from the Astoria Formation, Oregon, and a classification of the Otariidae (Mammalia: Carnivora). Contributions in Science, Natural History Museum of Los Angeles County 403: 1-26.

22. Barnes LG (1992) A new genus and species of Middle Miocene enaliarctine pinniped (Mammalia, Carnivora, Otariidae) from the Astoria Formation in coastal Oregon. Contributions in Science, Natural History Museum of Los Angeles County 431: 1-27.
